# Dendritic cell-expressed common gamma-chain recruits IL-15 for trans-presentation at the murine immunological synapse

**DOI:** 10.12688/wellcomeopenres.14493.2

**Published:** 2018-10-17

**Authors:** Chiara Beilin, Kaushik Choudhuri, Gerben Bouma, Dessislava Malinova, Jaime Llodra, David L. Stokes, Motumu Shimaoka, Timothy A. Springer, Michael L. Dustin, Adrian J. Thrasher, Siobhan O. Burns

**Affiliations:** 1 Molecular Immunology Unit, Institute of Child Health, University College London, London, WC1N 1EH, UK; 2 Program in Molecular Pathogenesis, Skirball Institute of Biomolecular Medicine, New York University, New York, NY, 10016, USA; 3 Program in Structural Biology, Skirball Institute of Biomolecular Medicine, New York University, New York, NY, 10016, USA; 4 Immune Disease Institute, Children's Hospital Boston, Boston, MA, 02115, USA; 5 Kennedy Institute of Rheumatology, Nuffield Department of Orthopedics, Rheumatology and Musculoskeletal Sciences, University of Oxford, Headington, OX3 7FY, UK; 6 Great Ormond Street Hospital for Children NHS Foundation Trust, London, WC1N 3JH, UK; 7 University College London Institute of Immunity and Transplantation, Department of Immunology, Royal Free London NHS Foundation Trust, London, NW3 2PF, UK

**Keywords:** interleukins, immunological synapse, immunodeficiency, trans-presentation, dendritic cells, lymphocytes

## Abstract

**Background:** Mutations of the common cytokine receptor gamma chain (γc) cause Severe Combined Immunodeficiency characterized by absent T and NK cell development. Although stem cell therapy restores these lineages, residual immune defects are observed that may result from selective persistence of γc-deficiency in myeloid lineages. However, little is known about the contribution of myeloid-expressed γc to protective immune responses.  Here we examine the importance of γc for myeloid dendritic cell (DC) function.

**Methods: **We utilize a combination of *in vitro* DC/T-cell co-culture assays and a novel lipid bilayer system mimicking the T cell surface to delineate the role of DC-expressed γc during DC/T-cell interaction.

**Results: **We observed that γc in DC was recruited to the contact interface following MHCII ligation, and promoted IL-15Rα colocalization with engaged MHCII. Unexpectedly, trans-presentation of IL-15 was required for optimal CD4+T cell activation by DC and depended on DC γc expression. Neither recruitment of IL-15Rα nor IL-15 trans-signaling at the DC immune synapse (IS), required γc signaling in DC, suggesting that γc facilitates IL-15 transpresentation through induced intermolecular *cis* associations or cytoskeletal reorganization following MHCII ligation.

**Conclusions: **These findings show that DC-expressed γc is required for effective antigen-induced CD4+ T cell activation. We reveal a novel mechanism for recruitment of DC IL-15/IL-15Rα complexes to the IS, leading to CD4+ T cell costimulation through localized IL-15 transpresentation that is coordinated with antigen-recognition.

## Introduction

Severe Combined Immunodeficiency (SCID) caused by deficiency of the common cytokine receptor gamma chain (γc) is characterized by defective T and NK cell development, resulting in life-threatening infections. Although the condition can be cured by bone marrow transplantation (BMT) or gene therapy, several long-term complications are seen; in particular a high incidence of severe cutaneous human papilloma virus (HPV) infection that suggests residual defects of immunity ^1– 3^. HPV susceptibility is not predicted by transplantation conditions or subsequent immune reconstitution but is curiously restricted to SCID resulting from mutations in γc or its signaling mediator Janus-associated kinase 3 (JAK3) and therefore appears to be related to the original genetic mutation.

HPV infections are limited to the epidermis suggesting persistent defects in the skin compartment, which could relate to keratinocytes or hematopoietic-derived immune cells. As many SCID patients receive BMT without any chemotherapy conditioning, B cell and myeloid lineages remain of host origin and therefore γc-deficient in the majority of cases ^1, 2^. This includes antigen-presenting dendritic cells (DC) derived from bone marrow, such as dermal (migratory) DC ^4^ and those that self-renew in tissues, such as epidermal Langerhans cells (LC). Although the mechanisms are poorly understood, LC and dermal DC are predicted to be important for regression of cutaneous HPV lesions through their role as potent skin antigen presenting cells for priming adaptive immune responses ^5, 6^. It is thought that CD4+ T cells also play a central role in anti-HPV immunity, as their presence at sites of HPV infection are predictive of clearance, while susceptibility to HPV infection is dramatically increased by CD4+ T cell immunodeficiency ^7– 11^. Since γc-deficiency in T cells is effectively corrected in SCID patients who have undergone BMT, we speculated that γc-deficient residual DC might be defective in priming antigen-specific CD4+ T cells in these patients, and hence might contribute to the observed impaired immunity to infection.

 As *ex vivo* isolation of primary LC and dermal DC populations in large numbers is technically challenging, we modeled DC γc-deficiency using monocyte-derived DC generated from the bone marrow of γc-deficient mice. While DC subsets differ in specific functions that likely relate to the particular requirements of their tissue environments, all myeloid-derived DC populations share prototypical features, including antigen uptake, presentation and T cell priming ^4^. DC/LC normally express several γc-containing cytokine receptors: specifically IL-2R, IL-4R, IL-15R and IL-21R that, upon binding their respective cytokines, regulate DC functions such as activation and cytokine release ^12^. In addition, DC-expressed IL-15R (and possibly IL-2R) regulates the function of other immune cells through the unusual mechanism of cytokine transpresentation that requires direct intercellular interaction ^13– 15^. In particular, transpresentation of IL-15 by DC is required for NK cell and memory CD8+ T cell activation and homeostasis ^13, 16^. Although several studies have shown that IL-15 enhances CD4+ T cell proliferation and is required for CD4+ memory homeostasis ^17– 22^ the importance of transpresentation for IL-15-dependent T-cell functions has not been clear until recently when effector CD4+ T-cell differentiation was shown to rely on transpresented rather than soluble IL-15 ^23^. To date, this has not been further detailed at a mechanistic level and a role for DC-mediated IL-15 transpresentation in CD4+ T-cell activation has not been documented.

In this study, we investigate the role of γc in DC function and identify a defect in the ability of γc-deficient DC to prime naïve CD4+ T cells. Using a novel supported planar bilayer system that mimics key molecular features of the T cell surface, we demonstrate that, independent of its signaling function, DC-expressed γc localises to the DC:T-cell contact interface following MHCII ligation and results in recruitment and colocalization of IL-15Rα with MHCII. We show that γc-deficiency in DC critically impairs IL-15Rα recruitment and IL-15 transpresentation to naïve CD4+ T cells at the immunological synapse, resulting in incomplete T cell activation. In light of these findings, we suggest a novel model for IL-15 transpresentation in which the DC-IS regulates co-stimulation of CD4+ T-cells during antigen-dependent priming.

## Methods

### Animals

Mice were C57BL/6 wild-type and OTII transgenic (OVA _323–339_ peptide (pOVA)/I-A ^b^-specific CD4+ T cells) (Charles River, Kent, UK). γc/Rag2 ^-/-^ mice (C57BL/6) were kindly provided by Dr. Colucci (Babraham Institute, Cambridge, UK). Male and female mice were housed in individually ventilated cages, up to 6 mice per cage with bedding changed twice weekly and sacrificed by exposure to a rising concentration of C0 _2_ at 8–12 weeks of age weighing approximately 25–30g. Bone marrow was extracted from tibia and femur bones. Work in mice was performed in an ethical manner according to UK Home Office regulations under project licence number PPL 70/7329.

### Cloning procedures

The lentiviral construct encoding γc ^WT^-GFP fusion protein (pLV-CMVEI.hIL2RG-SceI-EGFPds) was kindly provided by Nadine Dannemann, Toni Cathomen Lab, Hannover Medical School. A truncated γc ^Δc^-GFP was created by introduction of AgeI site by PCR with following primers: forward primer (GAAGACACCGACTCTAGAGCCACCATGTTG), reverse primer (CAACCGGTGGGCATCGTCCGTTCCAG). The PCR product was digested with XbaI and AgeI and religated into the original vector to create a γc ^Δc^-GFP fusion lacking 77 amino acids at the C terminus.

### Cell isolation and culture

Bone marrow-derived DC (BMDC) were generated, LPS-matured and OVA-pulsed as previously described ^24^. Briefly, bone-marrow (BM) cells were extracted from the femur and tibia of mice. To generate BMDC, BM cells were cultured over 7 days in RPMI medium 1640 supplemented with 10% fetal bovine serum (FBS) and 1% Penicillin/Streptomycin (Gibco) in the presence of 20 ng/ml GM-CSF (BioSource). For all experiments, BM DCs were CD11c selected using magnetic bead separation (Miltenyi Biotech). To induce DC activation, CD11c+ DCs were matured overnight with 100ng/ml LPS (Sigma). BMDC were blocked for 30 min at 37°C with anti-IL-15Rα (AF551) or isotype-matched controls (both R&D Systems, 20μg/ml unless otherwise stated). DC were nucleoporated with 5μg lentiviral plasmid DNA using the Amaxa Mouse Dendritic Cell Nucleofector Kit (Lonza). Unsorted cells were used for experiments. Splenic CD4+ T cells were isolated using a negative selection magnetic bead isolation kit (Miltenyi Biotech). For proliferation experiments, CD4+ T cells were labeled with 5μM CFSE dye for 20 min at 37°C then washed before co-culture.

### ELISA and flow cytometry assays

Supernatants of LPS-stimulated DC were assayed for IL-1β, IL-10, IL-12 using the Beadlyte ^®^ system (Millipore) and for IL-6 and TNF-α on ELISA (eBioscience). IL-2 secretion by CD4+ T cells was analysed with mouse IL-2 ELISA kit (R&D Systems). pSTAT5 assays were performed as previously described ^25^ using serum-starved CD4+ T cells co-cultured at 1:1 ratio for 10 min at 37°C with DC. Antibodies used for flow cytometry were against CD16/CD33 (2.4G2), CD86 (GL1), CD11c (HL3), CD4 (RM 4-5, SK3), I-A/I-E (2G9), pSTAT5 (pTyr694; clone 47) (all BD Biosciences), IL-15Rα (AF551) (R&D systems) and against γc (M-20) and IL-15 (H-114) (both from Santa Cruz Biotechnology). Apoptosis was assessed using the AnnexinV Apoptosis Detection Kit (BD Biosciences).

### Antigen uptake and presentation assays

Uptake and breakdown of DQ-OVA (self-quenched fluorescent conjugate of ovalbumin, Molecular Probes, Invitrogen), measured as emission of green fluorescence (515nm), were assessed as previously described ^24^. For measurement of antigen presentation, DC were matured overnight with LPS in the absence or presence of the indicated concentrations of Eα -GFP protein (kindly provided by Dr. Paul Garside, University of Glasgow). Eα peptide presentation was measured after 24hrs by flow cytometry. Briefly, cells were stained with antibodies against CD11c, IA/IE and the biotinylated Yae (specific for Eα ^52-68^ peptide presented on I-Ab) antibody (eBioscience) followed by streptavidin. DC were gated as CD11c ^+^IA/IE ^hi^ cells and presentation of Eα calculated as an index relative to DC matured in the absence of Eα -GFP (LPS only) using the following equation: 100 × (log ^Ea^/log ^LPS^) – log ^LPS^. For measurement of antigen presentation, DC were pulsed overnight with varying concentrations of OVA in the presence of LPS then co-cultured for 48hrs at a 1:5 ratio with BO17.4 hybridoma cells. IL-2 secretion by BO17.4 cells was measured by ELISA.

### Planar lipid bilayers

Liposome stocks containing DOPC, 25 mol% DGS-NTA and 2 mol% Cap-biotin (Avanti Polar Lipids) were prepared as described elsewhere ^26^. To make glass-supported planar bilayers for DC imaging, liposomes were mixed in appropriate ratios to produce DOPC bilayers with 0.01 mol% Cap-biotin and 12.5% DGS-NTA. Following washing with HBS containing 1% human serum albumin, 1mM Ca and 2mM Mg (HBS/HSA), bilayers were blocked with 5% Casein containing 100μM NiCl _2_, and incubated with 5μg/ml strepatavidin in HBS/HSA for 15min, and following washing, incubated for a further 30 min with a mixture of LFA-I domain-His6 (10μg/ml) and monobiotinylated anti-I-A/E Fab’ fragments (5ug/ml). Further details of anti-I-A/E, LFA and ICAM protein preparations are available in the Supplemental material). For imaging of OTII T cells, DOPC bilayers were prepared as above, containing 12.5% DOGS-NTA. ICAM-his12 and I-A ^b^/OVA-his12 were added to bilayers to yield densities of 300 mol/μm ^2^ and 100 mol/μm ^2 ^respectively. DOPC liposomes containing CD80 were incorporated at 200 mol/μm ^2^. Soluble IL-15/IL-15Rα with a C-terminal 6-histidine tag (eBioscience) was incorporated (2μg/ml) as indicated.

### Microscopy

TIRF imaging was performed using a Nikon Ti microscope equipped with a 100x Nikon TIRF objective, NA 1.49. Cells interacting with bilayers were fixed with 2% PFA; permeabilised with 0.1% saponin and quenched with 50mM glycine; blocked and stained with pSTAT5 (D47E7) (Cell Signaling) or IL-15Rα (H-107) (Santa Cruz Biotechnology). Secondary antibodies used were anti-rabbit AlexaFluor488 (Molecular Probes, Invitrogen). Measurement of labeled molecules was achieved by determining fluorescence intensity within regions of cell contact identified either using a threshold on TCR intensity (pSTAT5) or by the IRM channel (IL-15Rα). For analysis of MHCII and GFP accumulation at DC interfaces, fluorescence intensities were acquired at 4 frames/min over 25-min. Data were analysed with the Metamorph and ImageJ software. Please see Supplementary File 1 for more details on imaging methodology.

### Imaging of DC on lipid bilayers

Tracking of DC by confocal imaging was performed at 37°C in a heated environmental chamber. LPS/OVA-stimulated DC were introduced into flow-cells and areas of bilayers, selected at random, imaged for 37–45 min at 15 sec intervals. DIC and reflection (IRM) channels were recorded (+/- AF568 fluorescence) using appropriate laser excitation and emission filters. Cells were tracked manually in ImageJ software using cell nuclei in DIC images as a position reference. For quantitation of fluorescence intensities at DC interfaces with planar bilayers by TIRFM, cell contacts in the central region of the TIRF field, which is more evenly illuminated than the edges, were analyzed to minimize variations due to the inherent curvature of TIRF mode illumination. To estimate the extent to which variations in TIRF illumination contributed to the observed differences in measurements of specific fluorescence, the anti-MHC II Fab’ AF568 fluorescence intensity in bilayer regions immediately adjacent to DC interfaces was measured for all interfaces from which IL-15Rα fluorescence intensity was quantitated. Since non-interface anti-MHC II Fab’ AF568 is evenly distributed on bilayers, its fluorescence effectively represents laser excitation, in TIRF mode, within the imaging field. The morphology of the TIRF field was comparable between fluorescence channels. This baseline anti-MHC II Fab’ AF568 fluorescence was used to estimate the contribution of inter-sample (between γc ^-/-^ and ** WT DC samples) variation in TIRF illumination in interface fluorescence intensity measurements. Colocalization between engaged MHC II and IL-15Rα at DC interfaces was measured using Pearson correlation coefficient (PCC). To rule out spurious differences in PCC due to lower IL-15Rα fluorescence intensity at γc ^-/-^ DC interfaces, PCC between MHC II and IL-15Rα was calculated for a subset of γc ^-/-^ and WT interfaces with comparable IL-15Rα fluorescence intensity.

### Intracellular Ca ^2+ ^imaging

Bilayers containing LFA-1 Iα with or without anti-MHCII Fab’ fragments were made in FCS II flow cells as described above. Prior to introduction of DCs, flow cells were equilibrated to 37°C in the heated environmental of an LSM510 confocal microscope. DCs were loaded with 3 μM Fluo-4 AM (Invitrogen) for 20 min in serum free media, washed, and incubated for a further 20 min in complete cell culture media. Cells were subsequently washed, resuspended in HBS/HSA and introduced into flow chambers for confocal imaging using a 20x, NA 0.75 air objective, and wide confocal iris settings. All imaging was performed at 37°C, and images acquired for Fluo-4 and DIC channels every 15 seconds for ^~^25 minutes. Cell tracking and mean Flou-4 flourescence was measured using ImageJ.

### Statistics

*Prism v.5* (GraphPad Software) was used for statistical analysis. This included two-tailed Student’s t-test with 95% confidence bounds, one-way ANOVA (with Bonferroni’s correction for multiple comparison), Gaussian curve-fitting was performed with single, bimodal, and trimodal model parameters.

## Results

### γc ^-/-^ DC fail to trans-present IL-15 during antigen specific activation of naïve CD4+ T cells

**Figure 1.**
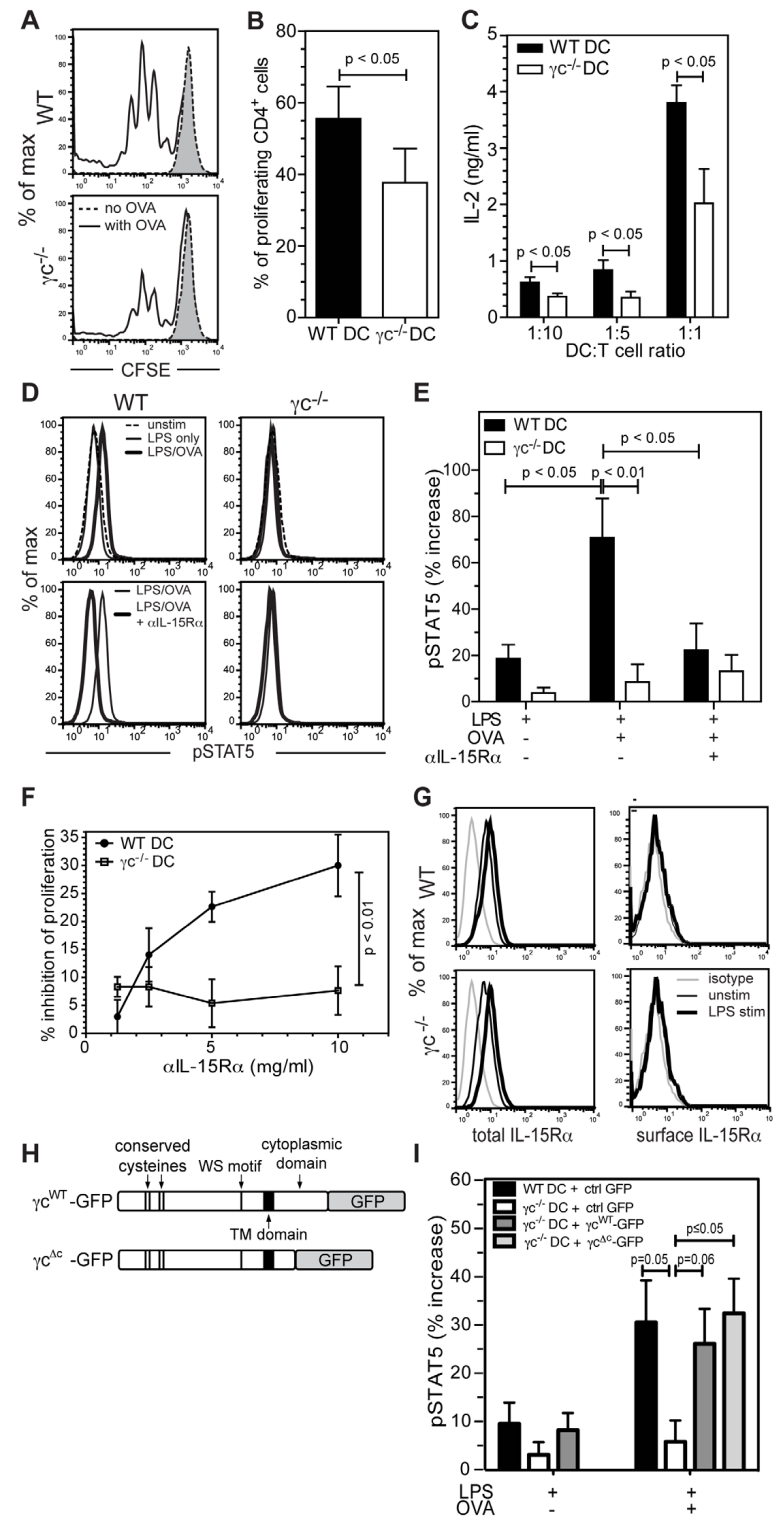
γc ^-/-^ DC fail to transpresent IL-15 to CD4+ T cells during antigen-specific priming. ( **A**) Representative plots showing CFSE dilution in CD4+-gated OTII T cells after co-culture with LPS-matured OVA-pulsed DC for 72 hrs. Grey dotted histograms represent CD4+ T cells cultured with DC in the absence of antigen. ( **B**) Quantification of T cell proliferation shown in A. ( **C**) IL-2 release by CD4+ T cells incubated with OVA-pulsed DC at the indicated ratios for 72hrs. ( **D**) pSTAT5 induction in CD4+ T cells following a 10 minute incubation with DC, either untreated or stimulated overnight with LPS ± OVA. DC were blocked with anti-IL-15Rα or isotype control. ( **E**) Increase in pSTAT5 fluorescence ( **D**), compared to unstimulated control. ( **F**) Inhibition of CD4+ T cell proliferation after co-culture for 72hr with OVA-pulsed DC pre-treated with anti-IL-15Rα (relative to isotype-matched control antibody treatment). ( **G**) Total and surface IL-15Rα expression (FAB551F) on CD11c gated cells. ( **H**) Schematic of construct encoding full length (γc ^WT^-GFP) and truncated (γc ^Δc^-GFP) γc attached to GFP. ( **I**) Increase in pSTAT5 levels, compared to unstimulated control, in CD4+ T cells following incubation with DC ±OVA, transfected with γc ^WT^-GFP, γc ^Δc^-GFP or ctrl GFP. *P* values, *t*-test ( **B**, **C**, **I**) ; one-way ANOVA ( **E**); linear regression (p value tests for significant difference between the slope of each line) ( **F**).

To investigate the role of γc in DC function, we generated conventional bone marrow-derived DC (BMDC) from γc-deficient (γc ^-/-^) mice ^27^. These mice also lack lymphoid-restricted recombinase activating gene 2 (RAG 2) by genetic modification, to eliminate low levels of persisting T cells seen in γc single knockout strains ^28^. Deletion of RAG 2 does not impair the function of GMCSF-derived BMDC ^29^ consistent with a lack of expression of VDJ rearrangement genes and RAG transcripts in conventional DC ^30^. Assessment of *ex vivo* splenic DC demonstrated low total DC numbers ( Figure S1A), as previously described for other T-lymphopenic mice ^31^, but comparable frequencies of CD11c+ CD11b+ and CD11c+ CD8α+ conventional DC and CD11c+ B220+ plasmacytoid DC subsets ( Figure S1B,C) suggesting that, *in vivo*, γc is dispensable for DC differentiation. As expected, BMDC derived from γc ^-/-^ mice completely lacked γc protein expression and γc-dependent cytokine signaling ( Figure S2A,B) but expressed the DC marker CD11c, MHCII and the costimulatory molecules CD86 and CD80 at levels comparable with WT DC both in the immature state and following LPS-induced maturation ( Figure S2C–E). To test whether γc ^-/-^ DC support normal antigen-mediated priming of T cells, DC were pulsed with whole ovalbumin (OVA) that is internalized, processed and presented on the surface of DC as a peptide antigen (pOVA) in complex with the class II MHC molecule I-A ^b^. When co-cultured with OTII CD4+ T cells, transgenic for a TCR recognising pOVA/I-A ^b^, OVA–pulsed γc ^-/-^ DC induced a moderate but significantly lower level of T cell proliferation than WT DC (p≤0.05, Figure 1A,B) and markedly reduced IL-2 secretion (p≤0.05, Figure 1C). As previously described ^32^, under these conditions, IL-2 release by DC was negligible ( Figure S2F) indicating that the impairment was due to defective T cell activation. Taken together, these data show that DC-expressed γc is required for full activation of antigen-specific CD4+ T cells.

Our findings were not due to impaired antigen uptake, processing or presentation of surface MHC/antigen complexes as γc ^-/-^ DC were as efficient as WT DC at internalising and processing DQ™ ovalbumin ( Figure S2G) and at processing and presenting the model Eα antigen ( Figure S2H,I). Furthermore, OVA-pulsed mature γc ^-/-^ and WT DC induced similar levels of IL-2 release from the B017.4 T cell hybridoma (which expresses the OTII TCR and is less dependent on costimulation) ( Figure S2J), demonstrating that pOVA presentation by surface MHC molecules was functionally similar between WT and γc ^-/-^ DC. Together, these data demonstrate that the observed defects in γc ^-/-^ DC mediated CD4+ T cell activation are not explained by defective antigen handling. As mature γc ^-/-^ and WT DC released similar levels of pro-inflammatory cytokines such as IL-1-β, IL6, IL-12, IFN-α and TNF-α ( Figure S2K), we reasoned that the observed defect of T-cell activation was due to a contact dependent rather than a soluble messenger mechanism.

As it is known that optimal naïve T cell activation depends on stable adhesion to DC ^33^, we investigated whether γc-deficiency impaired T-DC intercellular adhesion. Both conjugate-formation and redistribution of LFA-1 to the IS, a hallmark of T cell polarisation in response to antigen recognition ^34^, were preserved in T cells co-cultured with γc ^-/-^ DC ( Figure S3A–C). Taken together, these data demonstrate that the defective antigen-specific T cell priming observed in γc ^-/-^ DC is not due to impaired adhesion or LFA-1/ICAM-1 dependent T cell polarization. We further examined the fine-structure of the T-DC contact interface using transmission electron microscopy. Binding of TCR to pMHC occurs at, and stabilises, regions of close contact ( ^~^12 nm apart) between apposed membranes at T-DC interfaces, which are thought to be critical for signaling ^35, 36^. Compared to WT DC interfaces, γc ^-/-^ DC formed a similar proportion of close contacts with T cells, interspersed between areas of greater membrane separation ( ^~^30–50 nm)( Figure S3D–G), demonstrating that antigen-induced close contacts were preserved in the absence of γc.

Since antigen presentation, adhesion, secretory, and canonical costimulatory functions appeared to be preserved in γc ^-/-^ DC, we considered other plausible defects in DC function that might account for incomplete T cell priming. One candidate for this is the delivery of IL-15 mediated stimulatory signals to T cells by transpresentation. While it is well established that DC transpresent IL-15 to CD8+ T cells and NK cells ^37, 38^, the role of IL-15 transpresentation in CD4+ T cell activation has only begun to be explored ^23^. To establish whether IL-15 transpresentation occurs during DC-CD4+ T cell interactions, we analysed STAT5 activation in OTII T cells after 10 minute co-culture with LPS-matured WT DC. We observed that significant induction of STAT5 phosphorylation in CD4+ T cells occurred only when DC had been pre-loaded with antigen (p≤0.05, Figure 1D,E). Pre-treatment with an IL-15 blocking ( Figure 1D,E), but not an IL-2 blocking ( Figure S4A,B), monoclonal antibody abolished STAT5 phosphorylation in OTII T cells indicating that STAT5 activation occurs primarily through IL-15 transpresentation during DC-mediated priming of naïve CD4+ T cells in our experimental system.

Notably, antigen-pulsed γc ^-/-^ DC were severely compromised in their ability to activate STAT5 in OTII T cells, compared with WT DC ( Figure 1D,E), strongly implicating a role for γc in IL-15 transpresentation by DC. T cell proliferation induced by antigen-pulsed WT DC was also inhibited by IL-15Rα blockade in a dose-dependent manner. Consistent with the notion that naïve CD4+ T cell priming by γc ^-/-^ DC is compromised primarily due to defective IL-15 transpresentation, the reduced antigen-specific proliferative response of naïve CD4+ T cells to γc ^-/-^ DC was not further affected by IL-15Rα blockade (p≤0.01, Figure 1F and Figure S4C). The observed differences in T cell proliferation were not attributable to differential post-activation T cell viability ( Figure S4D), or IL-15Rα expression, as both total and surface levels of IL-15Rα were comparable between WT and γc ^-/-^ DC ( Figure 1G). Levels of total and surface IL-15 available for transpresentation were also unaffected by absence of DC-γc ( Figure S4E).

To more definitely establish whether γc expression in DC was necessary for IL-15 trans-signaling to T cells, we transfected γc ^-/-^ DC with constructs encoding either GFP alone, the full-length γc fused to GFP ^39^ (γc ^WT^-GFP), or a truncated γc (γc ^Δc^-GFP), that lacks 77 amino acids at the cytoplasmic carboxy-terminus, allowing surface expression but not signaling function ^40^ ( Figure 1H and Figure S4F,G). Expression of γc ^WT^-GFP or γc ^Δc^-GFP in γc ^-/-^ DC rescued STAT5 activation in OTII T cells, while expression of GFP alone had no effect ( Figure 1I and Figure S4H). Similar levels of GFP expression (≈35%) were obtained with all constructs ( Figure S4I).

**Figure 2.**
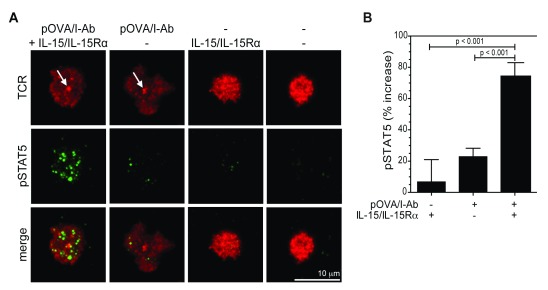
IL-15 mediated trans-signaling in CD4+ T cells requires TCR engagement. ( **A**) Representative TIRFM images of CD4+ T cells incubated on glass-supported planar bilayers containing ICAM-1, CD80, OVA _323-339_/I-A ^b ^and IL-15/IL-15Rα, as indicated. After 30 min incubation at 37°C in HBS/HSA buffer, cells were fixed and stained for TCR (red, Alexa Fluor 568) and phospho-STAT5 (pSTAT5) (green, Alexa Fluor 488) in PBS buffer. White arrows in the image panels indicate central accumulation of TCR at the T cells IS. ( **B**) Quantification of pSTAT5 fluorescence in A. Data are presented as percentage increase in pSTAT5 fluorescence, relative to unstimulated controls. Data are from 2 independent experiments (N=32-57) (mean ± S.E.M). *P* values, one-way ANOVA. Imaging was performed on a Nikon Ti microscope with a 100x TIRF objective, N.A. 1.49, controlled by Nikon Elements software. Fluorescence images were captured using an Ixon cooled EMCCD camera (512 x 512 pixels, Andor Technology). Mean fluorescence intensity at contact interfaces was quantified from 14 bit images using Metamorph software. Brightness and contrast are adjusted uniformly across image groups for clarity.

To confirm that IL-15 trans-presentation at the CD4+ T cell IS requires MHC:TCR engagement, we employed glass-supported planar lipid bilayers containing ICAM-1, CD80 and pOVA/I-A ^b ^ to recapitulate the essential features of an antigen-presenting surface suitable for naïve T cell stimulation, and incorporated IL-15/IL-15Rα complexes to mimic DC-mediated IL-15 transpresentation. Using this model system, we measured STAT5 phosphorylation as a marker of IL-2Rβ/γc mediated trans-signaling at the T cell IS by TIRFM. As expected, naïve OTII T cells formed a mature IS, at which TCR accumulated in a central supramolecular cluster (cSMAC) ^40^, only in response to pOVA/I-A ^b ^( Figure 2A, arrows). In keeping with our flow-cytometry results, IL-15/IL-15Rα did not activate STAT5 signaling in the absence of antigen, suggesting that TCR engagement is required for IL-15/IL-15Rα mediated trans-signaling in CD4+ T cells ( Figure 2A,B and Figure S5A,B). Despite dependence on TCR engagement for IL-15/IL-15Rα mediated STAT5 activation, PLCγ1 phosphorylation, which occurs downstream of TCR/CD28 signaling, and Akt phosphorylation, which is strongly induced by CD28 ligation, were not affected by IL-15 trans-signaling ( Figure S6A–D). Similarly, Zap-70 phosphorylation following TCR triggering was not affected by IL-15 trans-signaling, indicating minimal cross-talk between TCR/CD28 signals and the JAK/STAT pathway ( Figure S6E–G).

Taken together, these data demonstrate that DC transpresent IL-15 to CD4+ T cells by a mechanism that depends on MHCII/TCR ligation and DC-expressed γc. Surprisingly, γc signaling in DC was not required for IL-15 transpresentation, suggesting that γc facilitates IL-15 transpresentation through induced intermolecular *cis* interactions and/or cytoskeletal reorganization at the intramembrane or ectodomain level.

### Binding-induced clustering and accumulation of MHCII at the DC IS

**Figure 3.**
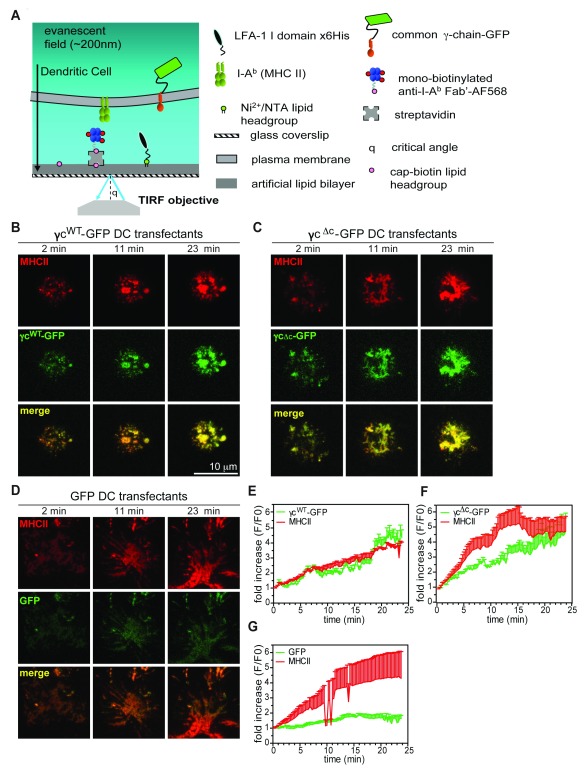
γc is recruited with MHCII at the DC IS. ( **A**) Schematic diagram of a glass-supported planar bilayer recapitulating a T cell surface. ( **B**– **D**) Representative TIRFM images demonstrating GFP (green) and MHCII (red, Alexa Fluor 568) accumulation over time at the contact interface of LPS/OVA stimulated γc ^-/-^ DC expressing γc ^WT^-GFP ( **B**), γc ^Δc^-GFP ( **C**) or GFP alone ( **D**), interacting on bilayers shown in A. ( **E**– **G**) Time course of GFP accumulation at the contact interface in DC transfected with γc ^WT^-GFP ( **E**)(N=6), γc ^Δc^-GFP ( **F**)(N=5) or GFP alone ( **G**)(N=5). Data represent mean fluorescence intensity (mean+S.E.M. are shown for clarity), normalized relative to values at initial point of contact by DC on bilayers (t=0). Live cells in HBS/HSA buffer were imaged in FCS2 flow chambers (Bioptechs) maintained at 37°C. Imaging was performed on a Nikon Ti microscope with a 100x TIRF objective, N.A. 1.49, controlled by Nikon Elements software. Fluorescence images were captured using an Ixon cooled EMCCD camera (512 x 512 pixels, Andor Technology). Mean fluorescence intensity at contact interfaces was quantified from 14 bit images using Metamorph software. Brightness and contrast are adjusted uniformly across image groups for clarity.

To investigate molecular events that follow MHC ligation at the DC IS at high spatial resolution, we developed a glass-supported bilayer system that recapitulates both adhesive and MHC-binding properties of the T cell surface ( Figure 3A). To ligate ICAM-1 on DC, we loaded bilayers with a C-terminally 6 histidine tagged inserted domain fragment of the LFA-1 α-subunit (αI) that is covalently locked in its high affinity conformation ^41^. To ligate MHCII, we generated C-terminally monobiotinylated Fab’ fragments ^42^ of an I-A/E specific monoclonal antibody (clone M5/114) to approximate TCR ectodomain size and valency (see Supplementary methods). These surrogate TCRs were attached to bilayers containing biotin headgroups *via* a streptavidin ‘bridge’, ensuring a uniform orientation that is favourable for MHC binding. Fragments were labelled with fluorophores (f/p ^^~^^3) to follow their recruitment and lateral reorganization upon binding to MHCII at the DC contact interface by confocal and TIRF microscopy.

Initial imaging by confocal microscopy revealed that WT DC exhibit a ‘crawling’ motility (mean velocity ^~^8 μm/min) on bilayers containing LFA-1 ( Movie S1, Figure S7A,C). Ligation of MHCII on WT DC led to an arrest in motility (mean velocity ^~^2.5 μm/min) and accumulation of engaged MHCII at the DC-bilayer interface ( Movie S2, Figure S7A,C). Although γc ^-/-^ DC migrated more slowly (mean velocity ^~^6 μm/min) compared to WT DC, ligation of MHCII led to a similar arrest in motility ( Movie S3, Figure S7B,D), indicating that MHCII ligation delivers a ‘stop’ signal to DCs, analogous to that in T cells following antigen recognition ^43^, that is not dependent on DC-γc expression. MHC II ligation does not lead to a rise in intracellular Ca ^2+^ levels in WT and γc ^-/-^ DC ( Movie S4,5 and Figure S7E–G), suggesting that, in contrast to antigen-induced T cell stopping, motility arrest following MHC II ligation in DC is not associated with Ca ^2+ ^signaling.

We next investigated the binding-induced organization of MHC II at the DC IS by total internal reflection fluorescence microscopy (TIRFM). Within seconds of contact with bilayers, ligated MHCII formed small clusters throughout the contact interface ( Figure 3B and Figure S7H), which were transported towards the center of the contact interface, presumably by interaction with the DC cytoskeleton ^24^ ( Movie S6,7). The extent of MHC accumulation at the contact interface was similar to that of WT DC ( Figure S7H,I).

### DC-expressed γc controls IL-15Rα recruitment to the IS

Mirroring MHC polarisation to the IS ^44, 45^, γc ^WT^-GFP and γc ^Δc^-GFP expressed in γc ^-/- ^DC were recruited to the contact interface, leading to ^~^4-fold enrichment of mean GFP fluorescence over 23 minutes ( Figure 3B,E and 3C,F). In contrast, GFP alone was not enriched at the contact interface over the same time course ( Figure 3D,G).

**Figure 4.**
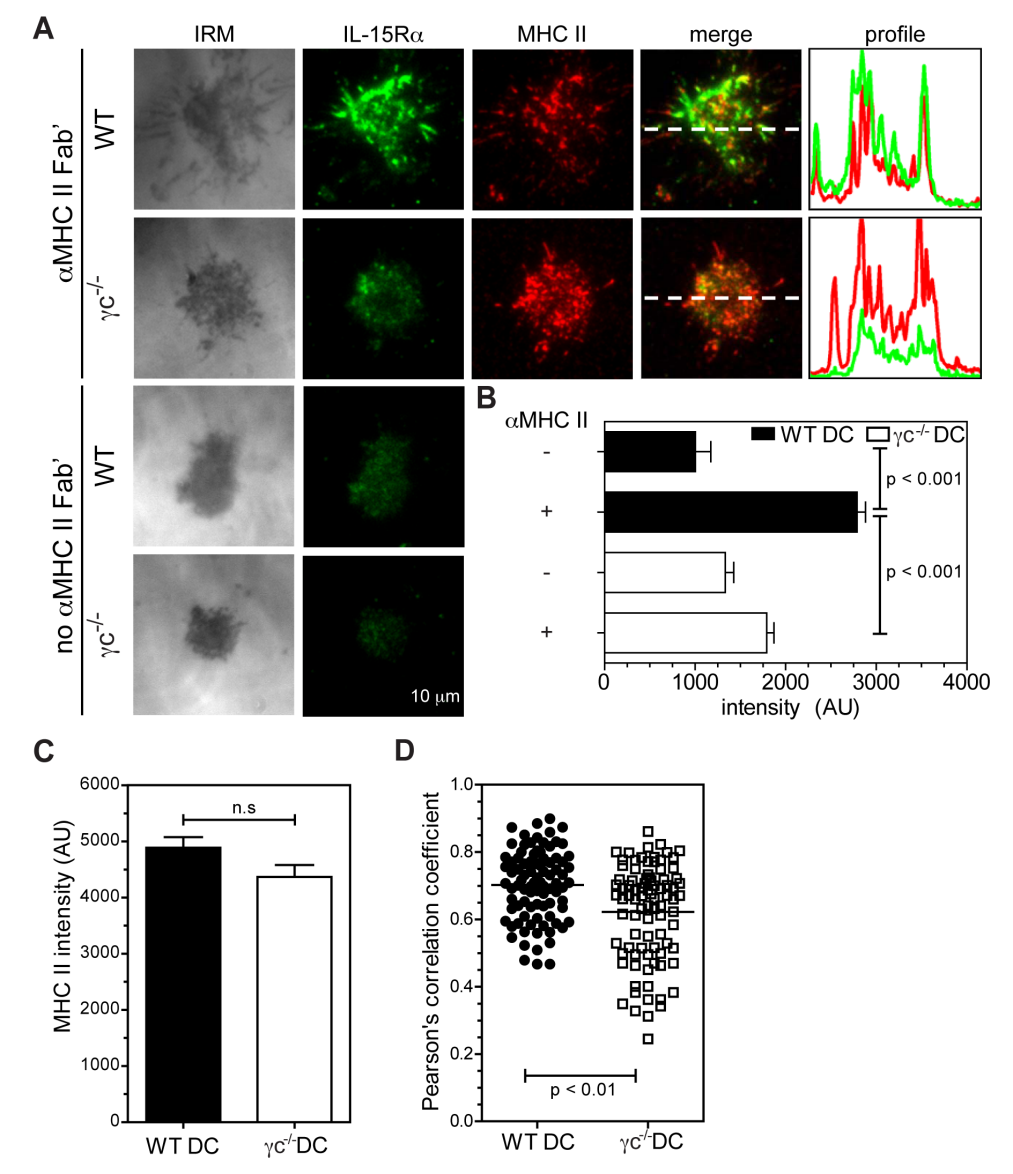
IL-15Rα is recruited to the DC interface and colocalizes with engaged MHCII. LPS/OVA stimulated DC were incubated on glass-supported planar bilayers containing LFA-1 αI ± I-A/E Fab’. After 15 min incubation at 37°C in HBS/HSA buffer, cells were fixed, permeabilized and stained for IL-15Rα (Alexa Fluor 488) in PBS buffer. ( **A**) Representative TIRFM images showing IL-15Rα and MHCII (Alexa Fluor 568) accumulation at contact interfaces. Fluorescence intensity profiles (right panels) indicate the distribution of IL-15Rα (green) and MHCII (red) along the dashed white lines (merge). ( **B**, **C**) Quantification in arbitrary units (AU) of mean IL-15Rα fluorescence ( **B**) and MHCII fluorescence ( **C**) at DC contact interfaces shown in A (N=89-90, mean ± S.E.M). ( **D**) Quantification of colocalization between MHCII and IL-15Rα at DC contact interfaces ( **A**), calculated as Pearson’s correlation coefficient (PCC). Imaging was performed on a Nikon Ti microscope with a 100x TIRF objective, N.A. 1.49, controlled by Nikon Elements software. Fluorescence images were captured using an Ixon cooled EMCCD camera (512 x 512 pixels, Andor Technology). Mean fluorescence intensity at contact interfaces was quantified from 14 bit images using Metamorph software. PCC was calculated for MHCII and IL-15Rα fluorescence channels using ImageJ software. Brightness and contrast are adjusted uniformly across image groups for clarity.

**Figure 5.**
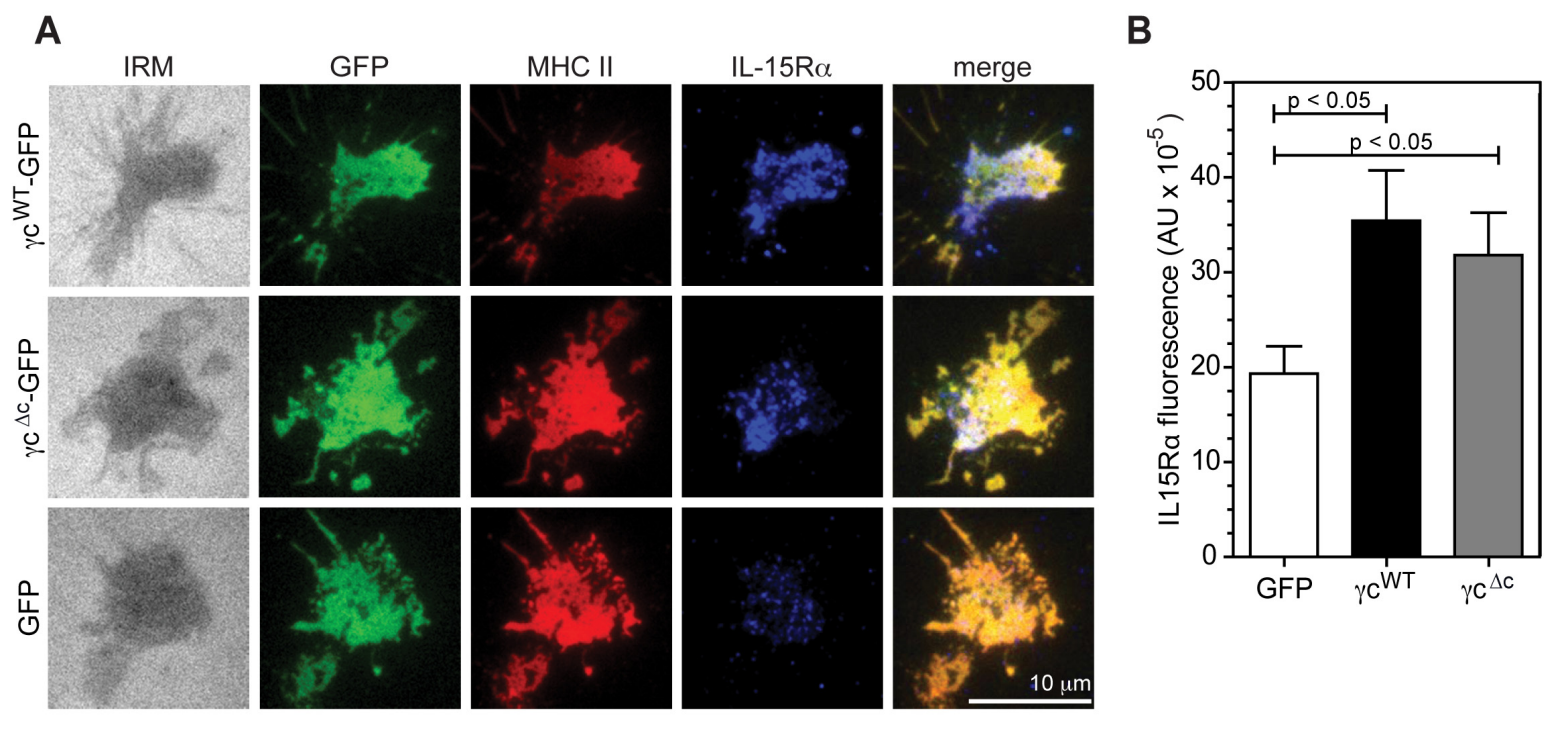
γc is required for IL-15Rα recruitment to the DC IS. ( **A**) Representative TIRFM images showing GFP, MHCII (Alexa Fluor 568) and IL-15Rα (Alexa Fluor 633) accumulation at contact interfaces of γc ^-/-^ DC, transfected with γc ^WT^-GFP, γc ^Δc^-GFP or ctrl GFP. ( **B**) Quantification in arbitrary units (AU) of mean IL-15Rα fluorescence at contact interfaces shown in A (N=38-43, mean ± S.E.M). P values, one-way ANOVA. After incubation on bilayers at 37°C in HBS/HSA buffer, cells were fixed and permeabilized to stain for IL-15Rα in PBS buffer. Imaging was performed on a Nikon Ti microscope with a 100x TIRF objective, N.A. 1.49, controlled by Nikon Elements software. Fluorescence images were captured using an Ixon cooled EMCCD camera (512 x 512 pixels, Andor Technology). Mean fluorescence intensity at contact interfaces was quantified from 14 bit images using Metamorph software. Brightness and contrast are adjusted uniformly across image groups for clarity.

Since DC-expressed γc was critical for effective IL-15 transpresentation in co-culture assays, its recruitment and colocalization with MHCII at the DC IS suggested the possibility of a spatially regulated mechanism for transpresentation, in which γc coordinates recruitment of IL-15/IL-15Rα to the DC synapse following MHCII ligation. To test this hypothesis, we imaged WT DC on bilayers at an early time-point (15 minutes), in the presence or absence of surrogate TCRs (anti-I-A/E Fab’), and labelled IL-15Rα for TIRF imaging. When compared to bilayers containing only LFA-1 αI domain, ligation of MHCII induced almost 3-fold more IL-15Rα at the DC IS, demonstrating that MHCII engagement is sufficient to recruit IL-15Rα to the IS in WT DC ( Figure 4A,B). Strikingly, MHCII-induced IL-15Rα recruitment to the IS was severely compromised in γc ^-/-^ DC (p≤0.001, Figure 4A,B) while MHCII accumulation was relatively unaffected ( Figure 4C). These differences were not accounted for by variations in TIRF imaging conditions between samples ( Figure S8). The extent of colocalization between MHCII and IL-15Rα was also decreased in the absence of γc, suggesting that it promoted a closer association between engaged MHCII and IL-15Rα ( Figure 4D and Figure S9). Expression of γc ^WT^-GFP and γc ^Δc^-GFP in γc ^-/-^ DC also resulted in ^~^70% increase in IL-15Rα accumulation at the interface upon MHC II ligation, when compared to expression of GFP alone ( Figure 5A,B), indicating that γc signaling in DC was dispensable for IL-15Rα recruitment.

## Discussion

Patients with γc-deficient SCID remain susceptible to opportunistic HPV infections even when T cell function is restored by BMT. This raises the possibility that residual γc-deficient DC, which persist in the absence of myeloablative conditioning, might be ineffective in priming T cell immunity. To investigate this in a tractable model, we tested the ability of bone-marrow derived DC from γc knockout mice to activate normal naïve CD4+ T cells. We have identified defects in the ability of γc-deficient DC to activate antigen-specific CD4+ T cells, which could not be accounted for by a problem with DC maturation or antigen processing. Instead, our studies have revealed an unexpected requirement for IL-15 transpresentation in CD4+ T cell activation. Furthermore, we have identified a role for DC γc in the recruitment of IL-15Rα to the DC side of the immune synapse, which is critical for effective IL-15 transpresentation to CD4+ T cells, and is independent of γc signaling function. Therefore, our *in vitro* functional and imaging studies have revealed a mechanism that may account for a subset of immune dysfunction in γc-deficient myeloid cells. While these studies have generated new hypotheses that can be explored further in human DC, the finding that IL-15 transpresentation contributes to CD4+ T cell activation in a DC γc-dependent manner, extends our understanding of the costimulatory requirements for CD4+ T cell priming. High resolution imaging of DC using a planar bilayer model system has provided new perspectives on the active role of DC in IS formation, that we expect will be useful for further investigation of the DC IS.

Soluble IL-15 produced in DC binds effectively irreversibly (K _D_^~^10 ^-11^ M) ^46^ to co-expressed IL-15Rα within intracellular compartments, before trafficking to the DC cell surface, for transpresentation to T and NK cells expressing IL-2Rβ/γc heterodimers ^47, 48^. Signal transduction in T cells occurs through the cytoplasmic portions of IL-2Rβ/γc heterodimers, which are associated with Janus family tyrosine kinases JAK1 and JAK3. Assembly of the IL-15/IL-15R ternary complex in *trans* leads to activation of JAK1/3, and subsequent phosphorylation of IL-2Rβ/γc. This leads to recruitment and activation of signal transducer and activator of transcription 5 (STAT5) proteins ^12^. We have shown in functional studies that IL-15 transpresentation by DC to CD4+ T cells is critically dependent on DC-expressed γc. Our imaging studies demonstrate that MHCII ligation leads to γc dependent recruitment of IL-15Rα to the DC IS, where it colocalizes with engaged MHCII. Both IL-15Rα recruitment to the DC IS, and IL-15 mediated trans-signaling in CD4+ T cells, are restored in γc-deficient DC following re-expression of γc. Neither process appeared to depend on signaling function as truncation of the γc cytoplasmic tail was also effective in recruiting IL-15Rα. Curiously, transpresented IL-15 triggered STAT5 signaling in CD4+ T cells only when TCR was engaged. Although it has been previously been shown that blockade of the DC IL-2Rα reduces T-cell activation ^14^, we were unable to demonstrate a contribution of DC-mediated IL-2Rα transpresentation in our system leading us to conclude that IL-15 is the major cytokine transpresented by DC for T-cell priming.

A precise picture of the molecular dynamics and subunit stoichiometries, in cell membranes, of IL-15/IL-15Rα and its associated receptor subunits has not yet been established. However, elegant imaging studies of IL-15Rα in transformed and primary T cell lines have revealed considerable heterogeneity in subunit composition, and a far more diverse set of *cis* associations, than might be predicted by ‘affinity conversion’ or other assembly models ^49^. Of relevance to our findings, MHCII has been shown to associate with both IL-15Rα ^50^ and with γc ^51^. Our observations, that γc is recruited to the DC IS, plays a critical role in recruitment of IL-15Rα, and promotes greater colocalization between engaged MHCII and IL-15Rα, lead us to favor a molecular configuration on the DC cell surface in which IL-15Rα, γc, and MHCII exist as a loosely coupled molecular complex, that is consolidated by MHCII engagement. Since MHCII engagement leads to its clustering and dynamic transport, presumably by interaction with the DC cytoskeleton, MHCII-nucleated domains may serve as avidity-enhancing scaffolds, or platforms within liquid-ordered lipid domains ^52^ that stabilize the IL-15Rα-γc-MHCII trimolecular association.

**Figure 6.**
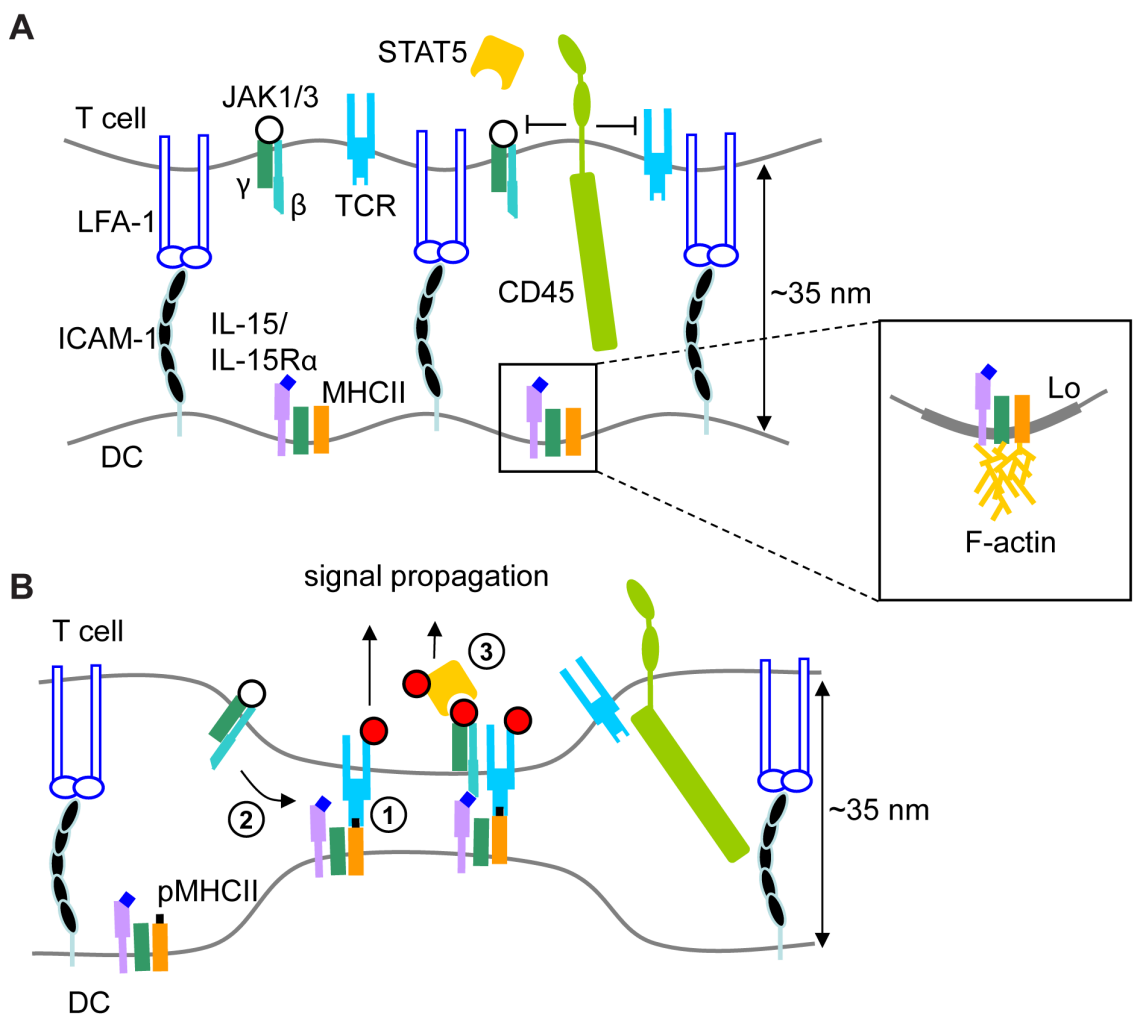
Model of γc-facilitated IL-15/IL-15Rα transpresentation to CD4+ T cells. ( **A**) In the absence of cognate peptide-MHC II (pMHCII), IL-2Rβ/γc cytokine receptor signaling in T cells is not initiated (open circles denote unphsophorylated IL-2Rβ/γc and associated JAK1/3), as these small receptors are likely positioned too far apart for stable binding of IL-15/IL-15Rα complexes on the DC surface. Inset depicts putative association of IL-15/IL-15Rα, MHCII and γc within liquid-ordered (Lo) lipid domains, and/or through cytoskeletal confinement. ( **B**) Engagement of pMHCII on DC (1) leads to γc-dependent recruitment IL-15/IL-15Rα to the contact interface, close to regions of bound pMHCII (2); this would position IL-15/IL-15Rα complexes on the DC surface at a distance compatible with binding IL-2Rβ/γc receptors in *trans* (2). Close contacts also exclude CD45, allowing stable phosphorylation (red circles) of both TCR and IL-2Rβ/γc receptors, permitting recruitment and phosphorylation of STAT5 (3) in the context of productive antigen recognition.

Taken together, our findings suggest a model of IL-15 transpresentation in which peptide/MHCII ligation by cognate TCR results in γc–mediated recruitment of IL-15Rα (in complex with IL-15) to the DC synapse, where it is positioned near sites of TCR engagement for binding in *trans* ( Figure 6A,B). Coupled delivery of IL-15 mediated costimulation with antigen recognition is consistent with the suggestion that close membrane apposition at the DC-T cell interface, determined by the (small) size of pMHC/TCR and accessory receptor complexes ( ^~^15 nm), may favor assembly of the *trans* IL-15/IL-15R ternary complex at DC-T-cell interfaces, since it is similar in size to TCR/pMHC complexes ^53^. TCR ligation of pMHC drives the formation of close contacts with APC, from which the large T cell surface phosphatase CD45, a key negative regulator of both TCR ^54^, and γc cytokine receptor-associated JAK signaling ^55^, is excluded ^36, 56^. Coupled (trans)presentation of pMHC and IL-15 at the DC-T cell IS may therefore allow spatially coordinated activation of the biochemically distinct TCR and JAK/STAT signaling pathways during antigen-specific priming of naïve T cells by DC.

## Data availability

Dataset 1: *Dendritic cell-expressed common gamma-chain recruits IL-15 for trans-presentation at the murine immunological synapse* is available from OSF: https://doi.org/10.17605/OSF.IO/YC7WS ^57^.

Data are available under the terms of the Creative Commons Zero “No rights reserved” data waiver (CC0 1.0 Public domain dedication).

Please see Supplementary File 4 for the data legend. Image data are available on request, see person to contact in Supplementary File 4.
