# Supplementary material for: Dendritic cell-expressed common gamma-chain recruits IL-15 for trans-presentation at the murine immunological synapse
Source: Wellcome Open Res. 2018 Oct 17;3:84. Originally published 2018 Jul 17. [Version 2] doi: 10.12688/wellcomeopenres.14493.2 (PMC6234741; doi:10.12688/wellcomeopenres.14493.2)
Supplement: Supplementary file 1 [file wellcomeopenres-3-16191-s0000.tgz › 7f01ec13-1a14-4a56-b132-86e0510553ca.docx]

**Supplementary File 1: Supplementary Methods**

**ELISA and flow cytometry assays**

Supernatants of LPS-stimulated DC were assayed for IL-1β, IL-10, IL-12 using the Beadlyte® system (Millipore) and for IL-6 and TNF-α on ELISA (eBioscience). Antibodies used for flow cytometry were against CD16/CD33 (2.4G2), CD86 (GL1), CD11c (HL3), I-A/I-E (2G9), (all BD Biosciences) and against γc (M-20) and IL-15 (H-114) ( both from Santa Cruz Biotechnology). Apoptosis was assessed using the AnnexinV Apoptosis Detection Kit (BD Biosciences).

# Cloning procedures

The lentiviral construct encoding γc^WT^-GFP fusion protein (pLV-CMVEI.hIL2RG-SceI-EGFPds) was kindly provided by Nadine Dannemann, Toni Cathomen Lab, Hannover Medical School. A truncated γc^Δc^-GFP was created by introduction of AgeI site by PCR with following primers: forward primer (GAAGACACCGACTCTAGAGCCACCATGTTG), reverse primer (CAACCGGTGGGCATCGTCCGTTCCAG). The PCR product was digested with XbaI and AgeI and religated into the original vector to create a γc^Δc^-GFP fusion lacking 77 amino acids at the C terminus.

**Immunoblotting**

Cell lysates of 10^6^ DC were separated by SDS-PAGE and blotted for γc using anti-IL-2Rγ (M-20) (Santa Cruz Biotechnology) or anti-GAPDH. pSTAT5 expression was determined by western blotting of whole-cell lysates with anti-pStat5 (Tyr694) (C71E5) and anti-STAT5 (3H7) (both from Cell Signaling Technology). Preparation of cell lysates and immunoblotting were performed as described previously[^1^](#_ENREF_1).

**Antigen uptake and presentation assays**

Uptake and breakdown of DQ-OVA (self-quenched fluorescent conjugate of ovalbumin, Molecular Probes, Invitrogen), measured as emission of green fluorescence (515nm), were assessed as previously described[^1^](#_ENREF_1). For measurement of antigen presentation, DC were matured overnight with LPS in the absence or presence of the indicated concentrations of Eα-GFP protein (kindly provided by Dr. Paul Garside, University of Glasgow). Eα peptide presentation was measured after 24hrs by flow cytometry. Briefly, cells were stained with antibodies against CD11c, IA/IE and the biotinylated Yae (specific for Eα^52-68^ peptide presented on I-Ab) antibody (eBioscience) followed by streptavidin. DC were gated as CD11c^+^IA/IE^hi^ cells and presentation of Eα calculated as an index relative to DC matured in the absence of Eα-GFP (LPS only) using the following equation: 100 x (log^Ea^/log^LPS^) – log^LPS^. For measurement of antigen presentation, DC were pulsed overnight with varying concentrations of OVA in the presence of LPS then co-cultured for 48hrs at a 1:5 ratio with BO17.4 hybridoma cells. IL-2 secretion by BO17.4 cells was measured by ELISA.

**Light microscopy**

Confocal imaging of DC-T cell conjugates was performed using a Zeiss LSM 510 confocal microscope (x63 objective, NA 1.4, Iris diameter set at 1 Airy unit). Cells were plated on polylysine-coated coverslips, fixed with 2% PFA, permeabilised with 0.1% saponin, quenched with 50mM glycine and blocked with 5% BSA. Cells were stained with anti-LFA-1 (YN1/1) followed by AF488 anti-rat Ab (Molecular Probes). Enrichment of LFA-1 at the T-DC interface was determined as described elsewhere[^2^](#_ENREF_2).

**Electron microscopy**

Conjugates of LPS-matured OVA-pulsed DC and CD4+ T cells were fixed in PBS containing 1% glutaraldehyde, 3% PFA, and 0.3% (w/v) tannic acid, for 1 hour. Samples were were dehydrated, embedded and sections as described elsewhere[^2^](#_ENREF_2). Intermembrane distances were measured as previously described[^2^](#_ENREF_2)^,^[^3^](#_ENREF_3) from high magnification images acquired by transmission electron microscopy (Phillips CM12) using a 4K x 2.7K pixel digital camera (Gatan).

**Protein preparation**

Anti-I-A/E monoclonal antibody M5/114[^4^](#_ENREF_4) was digested with pepsin to produce F(ab’)2 fragments. M5/114 F(ab’)2 was reduced in phosphate buffer containing 0.5mM 2-mercaptoethanol for 30min at 30°C, to yield Fab’-SH fragments. Following buffer exchange into PBS, Fab’ fragments were reacted with 5μg/ml maleimide-PEG2-biotin (Pierce) for 2hrs for conjugation to the Fab’ free sulfhydryl. Following buffer exchange into PBS bioinylated fragments were amine-conjugated to Alexa flourophore succinimidyl esters (AF568 or AF633). Dye to protein ratios were calculated by measuring UV-vis spectra peaks and using extinction coefficient for Fab’ at 280 nm (~70,000 M^-1^) and for flourophores at 280 nm and at their absorption peaks (AF568: 91,300 & 41,998; AF633: 100,000 & 51,000). ICAM-1-his12 and LFA-1 I domain-his6 were prepared as described elsewhere[^5^](#_ENREF_5)^,^[^6^](#_ENREF_6).

**Imaging of DC on lipid bilayers**

Tracking of DC by confocal imaging was performed at 37°C in a heated environmental chamber. LPS/OVA-stimulated DC were introduced into flow-cells and areas of bilayers, selected at random, imaged for 37-45 min at 15 sec intervals. DIC and reflection (IRM) channels were recorded (+/- AF568 fluorescence) using appropriate laser excitation and emission filters. Cells were tracked manually in Image J software using cell nuclei in DIC images as a position reference. For quantitation of fluorescence intensities at DC interfaces with planar bilayers by TIRFM, cell contacts in the central region of the TIRF field, which is more evenly illuminated than the edges, were analyzed to minimize variations due to the inherent curvature of TIRF mode illumination. To estimate the extent to which variations in TIRF illumination contributed to the observed differences in measurements of specific fluorescence, the anti-MHC II Fab’ AF568 fluorescence intensity in bilayer regions immediately adjacent to DC interfaces was measured for all interfaces from which IL-15Rα fluorescence intensity was quantitated. Since non-interface anti-MHC II Fab’ AF568 is evenly distributed on bilayers, its fluorescence effectively represents laser excitation, in TIRF mode, within the imaging field. The morphology of the TIRF field was comparable between fluorescence channels. This baseline anti-MHC II Fab’ AF568 fluorescence was used to estimate the contribution of inter-sample (between γc^-/-^ and WT DC samples) variation in TIRF illumination in interface fluorescence intensity measurements. Colocalization between engaged MHC II and IL-15Rα at DC interfaces was measured using Pearson correlation coefficient (PCC). To rule out spurious differences in PCC due to lower IL-15Rα fluorescence intensity at γc^-/-^ DC interfaces, PCC between MHC II and IL-15Rα was calculated for a subset of γc^-/-^ and WT interfaces with comparable IL-15Rα fluorescence intensity.

**Intracellular Ca^2+^ imaging**

Bilayers containing LFA-1 Iα with or without anti-MHCII Fab’ fragments were made in FCS II flow cells as described above. Prior to introduction of DCs, flow cells were equilibrated to 37°C in the heated environmental of an LSM510 confocal microscope. DCs were loaded with 3 μM Fluo-4 AM (Invitrogen) for 20 min in serum free media, washed, and incubated for a further 20 min in complete cell culture media. Cells were subsequently washed, resuspended in HBS/HSA and introduced into flow chambers for confocal imaging using a 20x, NA 0.75 air objective, and wide confocal iris settings. All imaging was performed at 37°C, and images acquired for Fluo-4 and DIC channels every 15 seconds for ~25 minutes. Cell tracking and mean Flou-4 flourescence was measured using ImageJ.

**References**

1. Bouma G, Mendoza-Naranjo A, Blundell MP, et al. Cytoskeletal remodeling mediated by WASp in dendritic cells is necessary for normal immune synapse formation and T-cell priming. *Blood*. 2011;118(9):2492-2501.

2. Choudhuri K, Wiseman D, Brown MH, Gould K, van der Merwe PA. T-cell receptor triggering is critically dependent on the dimensions of its peptide-MHC ligand. *Nature*. 2005;436(7050):578-582.

3. Milstein O, Tseng SY, Starr T, et al. Nanoscale increases in CD2-CD48-mediated intermembrane spacing decrease adhesion and reorganize the immunological synapse. *The Journal of biological chemistry*. 2008;283(49):34414-34422.

4. Germain RN, Bhattacharya A, Dorf ME, Springer TA. A single monoclonal anti-Ia antibody inhibits antigen-specific T cell proliferation controlled by distinct Ir genes mapping in different H-2 I subregions. *Journal of immunology*. 1982;128(3):1409-1413.

5. Schubert DA, Gordo S, Sabatino JJ, Jr., et al. Self-reactive human CD4 T cell clones form unusual immunological synapses. *The Journal of experimental medicine*. 2012;209(2):335-352.

6. Luo BH, Takagi J, Springer TA. Locking the beta3 integrin I-like domain into high and low affinity conformations with disulfides. *The Journal of biological chemistry*. 2004;279(11):10215-10221.
