# Supplementary material for: Dendritic cell-expressed common gamma-chain recruits IL-15 for trans-presentation at the murine immunological synapse
Source: Wellcome Open Res. 2018 Oct 17;3:84. Originally published 2018 Jul 17. [Version 2] doi: 10.12688/wellcomeopenres.14493.2 (PMC6234741; doi:10.12688/wellcomeopenres.14493.2)
Supplement: Supplementary file 4 [file wellcomeopenres-3-16191-s0003.tgz › c9d05c09-7629-402b-a3a6-ba948069c7f3_Supplementary_file_4.docx]

**Main Figures**

| Figure | Short title | Legend |
| --- | --- | --- |
| 1A | Analysis of DC-mediated T cell priming – CFSE dilution | DC type: WT, KO (γc-/- DCs)  DC: T cell ratios: hi (1:1), med (1:5) and low (1:10)  DC treatment: LPS only, OVA (LPS+OVA)  Control: T cells only  Stains: CFSE (FL1), 7AAD  Software used: FlowJo |
| 1B | Analysis of DC-mediated T cell priming – CFSE dilution | DC: T cell ratios analysed: 1:10  Software used: GraphPad Prism |
| 1C | Analysis of DC-mediated T cell priming – IL-2 release | Software used: GraphPad Prism |
| 1D | Intracellular detection of pSTAT5 in CD4+ T cells - IL-15Rα block | DC type: WT, KO (γc-/- DCs)  DC treatment: LPS (LPS only), OVA (LPS+OVA), ISO (isotype control PE), BLOCK (anti-IL-15Rα)  Stains: CD11c APC, CD4 PE-Cy7, pSTAT5 PE  Software used: FlowJo |
| 1E | Intracellular detection of pSTAT5 in CD4+ T cells following incubation with DCs | Software used: GraphPad Prism |
| 1F | Analysis of DC-mediated T cell priming – blocking of anti-IL-15Rα on DCs | Software used: GraphPad Prism |
| 1G | Intracellular IL-15 staining | DC type: WT, KO (γc-/- DCs)  DC treatment: unstimulated, + (LPS)  Stains: CD11c APC, IL-15Ra FITC, isotype control (FITC)  Software used: FlowJo |
| 1G | Surface IL-15 staining | DC type: WT, KO (γc-/- DCs)  DC treatment: unstimulated, + (LPS)  Stains: CD11c APC, IL-15Ra FITC, isotype control (FITC)  Software used: FlowJo |
| 1I | Intracellular detection of pSTAT5 in CD4+ T cells following incubation with transfected DCs | DC type: WT, KO GFP (γc-/- DC transf with ctrl GFP), KO gc-GFP (γc-/- DC tranf with γc^WT^-GFP), KO mut gc-GFP (γc-/- DC tranf with (γc^Δc^-GFP).  DC treatment: -LPS, +LPS, +LPS+OVA  Software used: GraphPad Prism + Excel (note- MFI were normalized within experiments with unstimulated controls) |

| Figure | Short title | Legend |
| --- | --- | --- |
| 2A | Trans-presented IL-15 induces pSTAT5 in T cell immunological synapse. | Image data- contact  michael.dustin@kennedy.ox.ac.uk |
| 2B | Trans-presented IL-15 induces pSTAT5 in T cell immunological synapse. | OTII 112010.pzf  Software used: GraphPad Prism |

| Figure | Short title | Legend |
| --- | --- | --- |
| 3A | Schematic of experiment to test recruitment of γc to dendritic cell immunological synapse by IL-15 | No data |
| 3BCD | Recruitment of γc to dendritic cell immunological synapse by IL-15 | Image data- contact  michael.dustin@kennedy.ox.ac.uk |
| 3EFG | Recruitment of γc to dendritic cell immunological synapse by IL-15 | CgMut_GFP MHCII vs time.xls  Cg-GFP MHC vs time.xls  gcMut-GFP MHCII vs time.pzf  GFP MHCII vs time.pzf  Software used: GraphPad Prism and Microsoft Excel |

| Figure | Short title | Legend |
| --- | --- | --- |
| 4A | γc dependent recruitment of IL-15Rα to sites of MHC engagement | Images data- contact  michael.dustin@kennedy.ox.ac.uk |
| 4BC | γc dependent recruitment of IL-15Rα to sites of MHC engagement | IL-15Ra DC quant.pzf  IL-15Ra DC quant dot plots.pzf  Software used: GraphPad Prism |
| 4D | γc dependent IL-15Rα co-localization with MHC class II in model DC immunological synapse | il15 mhc pearsons.pzf  Software used: GraphPad Prism |

| Figure | Short title | Legend |
| --- | --- | --- |
| 5A | IL-15Rα recruitment to the DC immunological synapse | Image data- contact  michael.dustin@kennedy.ox.ac.uk |
| 5B | Analysis of IL-15Rα recruitment to the DC immunological synapse | IL15r rescue.pzf  Software used: GraphPad Prism |

**Supplementary Figures**

| Figure | Short title | Legend |
| --- | --- | --- |
| S1A | Frequency of γc-/- splenic DC | Image data- contact  siobhan.burns@ucl.ac.uk |
| S1B | Phenotype of γc-/- splenic DC | Image data- contact  siobhan.burns@ucl.ac.uk |
| S1C | Phenotype of γc-/- splenic DC | Image data- contact  siobhan.burns@ucl.ac.uk |

| Figure | Short title | Legend |
| --- | --- | --- |
| S2A | Absence of γc protein in knock out DC | Image data- contact  siobhan.burns@ucl.ac.uk |
| S2B | Absence of IL-21 induced pSTAT5 in  γc -/- DC | Image data- contact  siobhan.burns@ucl.ac.uk |
| S2C | Surface staining for CD11c | Image data- contact  siobhan.burns@ucl.ac.uk |
| S2D | Surface staining for activation markers | DC type: WT, KO (γc-/- DCs)  DC treatment: unstimulated, LPS-stimulated  Stains: CD11c APC, CD86 FITC, MHC II PE, isotype control (FITC), unstained  Software used: FlowJo |
| S2E | Surface staining for activation markers | Image data- contact  siobhan.burns@ucl.ac.uk |
| S2F | DC-induced IL-2 release | Software used: GraphPad Prism |
| S2G | Uptake and breakdown of DQ-OVA by LPS-matured DCs | DC type: WT, KO (γc-/- DCs)  DC treatment: LPS-stimulated  Controls: no cells |
| S2H | Normal antigen processing in γc-/- DC | Contact:  siobhan.burns@ucl.ac.uk |
| S2I | Normal antigen processing in γc-/- DC | Contact:  siobhan.burns@ucl.ac.uk |
| S2J | IL-2 production by BO17.4 hybridoma | DC type: WT, KO (γc-/- DCs)  DC treatment: LPS/OVA-stimulated  Software used: GraphPad Prism |
| S2K | Cytokine release by DC - Luminex | DC type: WT, KO (γc-/- DCs)  DC treatment: LPS-stimulated  Software used: GraphPad Prism |

| Figure | Short title | Legend |
| --- | --- | --- |
| S3A | Conjugate formation between DDAO-labelled CD4+ T cells and CFMDA-labelled DC | DC type: WT, KO (γc-/- DCs)  DC treatment: LPS only, OVA (LPS+OVA+ isotype control), LPS OVA BLOCK (LPS+OVA+anti-ICAM-1)  Stains: CFMDA (FL1), DDAO (FL4H)  Software used: FlowJo  Analysis of multiple experiments - software used: GraphPad Prism |
| S3B | Normal immunological synapse formation by γc-/- DCs | Image data- contact  siobhan.burns@ucl.ac.uk |
| S3C | Quantification of T cell LFA-1 enrichment at the IS | DC type: WT, KO (γc-/- DCs)  DC treatment: LPS only, OVA (LPS+OVA+ isotype control)  Software used: GraphPad Prism |
| S3D | Transmission electron microscopy of T cells-DC immunological synapse. | Image data- contact  michael.dustin@kennedy.ox.ac.uk |
| S3EF | Transmission electron microscopy based distance measurements between T cells and DC from in vitro conjugate experiments. | DC IM distances.xls  DC IM distances.pzf  DC-TC IM distances.pzf  DC IM distances fits.pzf  Software used: GraphPad Prism and Microsoft Excel |

| Figure | Short title | Legend |
| --- | --- | --- |
| S4A | Intracellular detection of pSTAT5 in CD4+ T cells - IL-2Rα block | DC type: WT, KO (γc-/- DCs) – 3 samples each  DC treatment: LPS (LPS only), OVA (LPS+OVA), ISO (isotype control PE), BLOCK (anti-IL-2Rα)  Controls: T cells only, DC only  Stains: CD4 PE-Cy7, pSTAT5 PE  Software used: FlowJo |
| S4C | Analysis of DC-mediated T cell priming – CFSE dilution - IL-15Rα block | DC type: WT, KO (γc-/- DCs)  DC treatment: cont  (LPS+OVA+isotype control), block  (LPS+OVA+anti- IL-15Rα (1.25, 2.5, 5, 10 ug/ml))  Control: no OVA stimulation  Stains: CFSE (FL1), CD4 APC  Software used: FlowJo |
| S4D | Quantification of cell viability in CD4+ T cells after co-culture with LPS-matured OVA-pulsed DC - IL-15Rα block | DC type: WT, KO (γc-/- DCs) – 3 samples each  DC treatment: (LPS+OVA+isotype control), block (LPS+OVA+anti-IL-15Rα  Control: T cells only  Stains: CD4 APC, AnnexinV-PI  Software used: FlowJo  Analysis of multiple experiments - software used: GraphPad Prism |
| S4E | Intracellular staining for IL-15 | DC type: WT, KO (γc-/- DCs)  DC treatment: unstimulated, + (LPS-stimulated)  Stains: CD11c APC, IL-15 FITC, CD11b PE, isotype control (FITC), unstained  Software used: FlowJo |
| S4E | Surface staining for IL-15 | DC type: WT, KO (γc-/- DCs)  DC treatment: unstimulated, + (LPS-stimulated)  Stains: CD11c APC, IL-15 FITC, CD11b PE, isotype control (FITC), unstained  Software used: FlowJo |
| S4F | yc staining on ED7R cells transfected with LV constructs | Cell type: ED7R (untransduced, transduced with ycWTGFP and γcΔc-GFP)  Stains: ycPE, unstained, PE stain only, GFP only  Software used: FlowJo |
| S4G | pSTAT5 staining on ED7R cells transfected with LV constructs | Cell type: ED7R (untransduced +/- IL-2), ED7Rgc (transduced with ycWTGFP +/- IL-2), ED7Rmutgc (transduced with γcΔc-GFP +/- IL-2  Stains: psTAT5PE, PE stain only, GFP only  Software used: FlowJo |
| S4H | pSTAT5 induction in CD4+ T cells following incubation with transfected DC | DC type: WT, KO (γc-/- DCs)  DC treatment: unstimulated, LPS (LPS-stimulated), transfected with gc (ycWTGFP) or mut (γcΔc-GFP)  Stains: CD4c PECy7, pSTAT5PE, T cells unstained, T cells only, pSTAT5 only  Software used: FlowJo |
| S4I | Levels of GFP expression obtained by nucleofecting DCs with γcWT-GFP, γcΔc-GFP or ctrl GFP | DC type: WT, KO (γc-/- DCs)  DC treatment: unstimulated, LPS (LPS-stimulated), transfected with gc (ycWTGFP) or mut (γcΔc-GFP)  Stains: CD4c PECy7, pSTAT5PE, T cells unstained, T cells only, pSTAT5 only  Software used: FlowJo |

| Figure | Short title | Legend |
| --- | --- | --- |
| S6A | Effect of IL-15 transpresentation on pPLCγ recruitment to immunological synapse | Image data- contact  michael.dustin@kennedy.ox.ac.uk |
| S6B | Quantification in AU of pPLCγ MFI at contact interfaces | Software used: GraphPad Prism |
| S6C | Effect of IL-15 transpresentation on pAkt recruitment to immunological synapse | Image data- contact  michael.dustin@kennedy.ox.ac.uk |
| S6D | Quantification in AU of pAkt MFI at contact interfaces | Software used: GraphPad Prism |
| S6E | Effect of IL-15 transpresentation on pZAP70 recruitment to immunological synapse | Image data- contact  michael.dustin@kennedy.ox.ac.uk |
| S6FG | Quantification in AU of pAkt MFI at contact interfaces | Quantication of integrated fluorescence intensity of ZAP-70 and TCR from image data. CB OTII pZAP70 quant.xls  Software used: Microsoft Excel |

| Figure | Short title | Legend |
| --- | --- | --- |
| S7A-D | Arrest of DC motility by anti-MHC- immunological synapse model. | DC motility.xls  DC motility.pzf  Software used: GraphPad Prism and Microsoft Excel |
| S7EFG | Ca^2+^ mobilization in response to anti-MHC- immunological synapse model. | DC Ca2 quant.xls  DC Ca2 response.pzf  Software used: GraphPad Prism and Microsoft Excel |
| S7H | Accumulation of anti-MHC class II in DC immunological synapse. | Image data- contact  michael.dustin@kennedy.ox.ac.uk |
| S7I | Accumulation of anti-MHC class II in DC immunological synapse. | dc live.xls  Software used: Microsoft Excel |

| Figure | Short title | Legend |
| --- | --- | --- |
| S9AB | Analysis of IL-15Rα accumulation in DC synapse and co-localization with MHC. | PNAS revision IL-15R Rr.xls  IL-15 PCC.pzf  Software used: GraphPad Prism and Microsoft Excel |

| Figure | Short title | Legend |
| --- | --- | --- |
| S8A | Quantification of MHC class II accumulation in DC immunological synapse model | Image data- contact  michael.dustin@kennedy.ox.ac.uk |
| S8B | Quantification of MHC class II accumulation in DC immunological synapse model | MHCII free bilayer.xls  Free MHC II.pzf  Software used: GraphPad Prism and Microsoft Excel |
