# Supplementary material for: Dendritic cell-expressed common gamma-chain recruits IL-15 for trans-presentation at the murine immunological synapse
Source: Wellcome Open Res. 2018 Oct 17;3:84. Originally published 2018 Jul 17. [Version 2] doi: 10.12688/wellcomeopenres.14493.2 (PMC6234741; doi:10.12688/wellcomeopenres.14493.2)
Supplement: Supplementary file 2 [file wellcomeopenres-3-16191-s0001.tgz › 2e35fac5-76aa-494d-bab0-c7b316927fdb_Supplementary_file_2.zip!/Figure_S1-9_revised.pdf.pdf]

Fig. S1

A

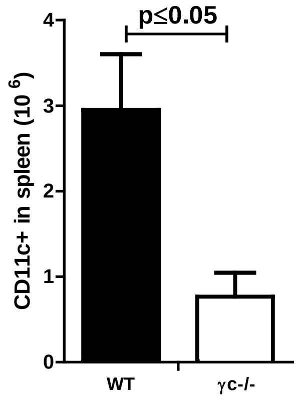

p≤0.05
CD11c+ in spleen (10 ^{6})
0
1
2
3
4
WT
γc-/-

B

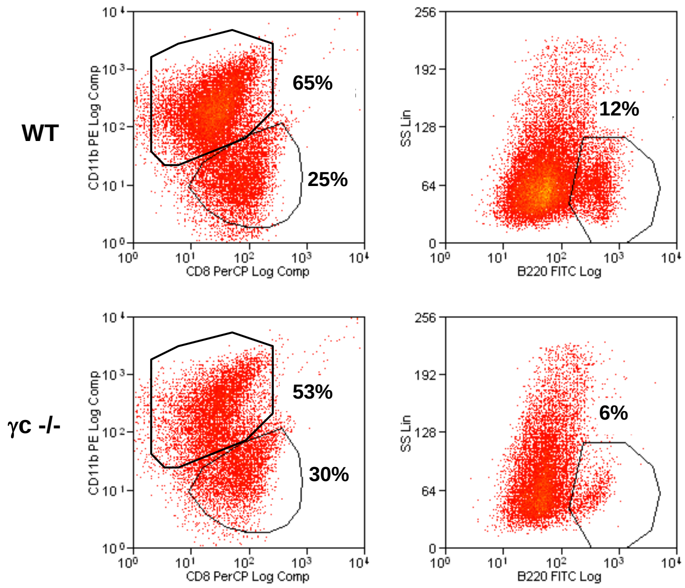

WT
γc -/-
65%
25%
12%
53%
30%
6%
CD11b PE Log Comp
CD8 PerCP Log Comp
SS Lin
B220 FITC Log

C

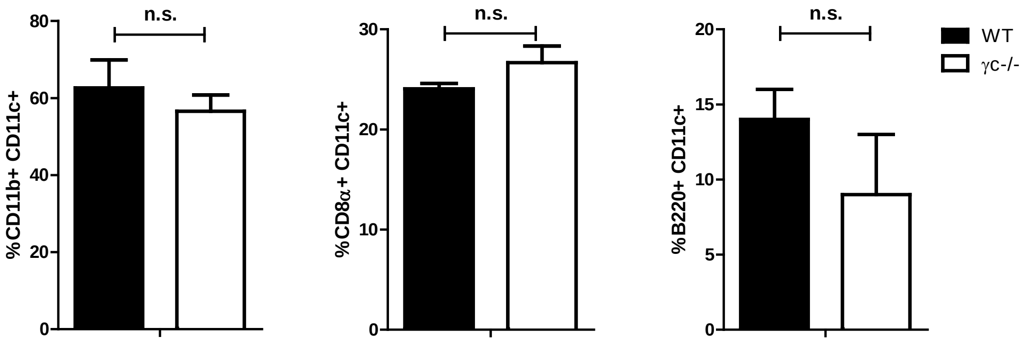

n.s.
n.s.
n.s.
%CD11b+ CD11c+
%CD8α+ CD11c+
%B220+ CD11c+
WT
γc-/-

Fig. S2

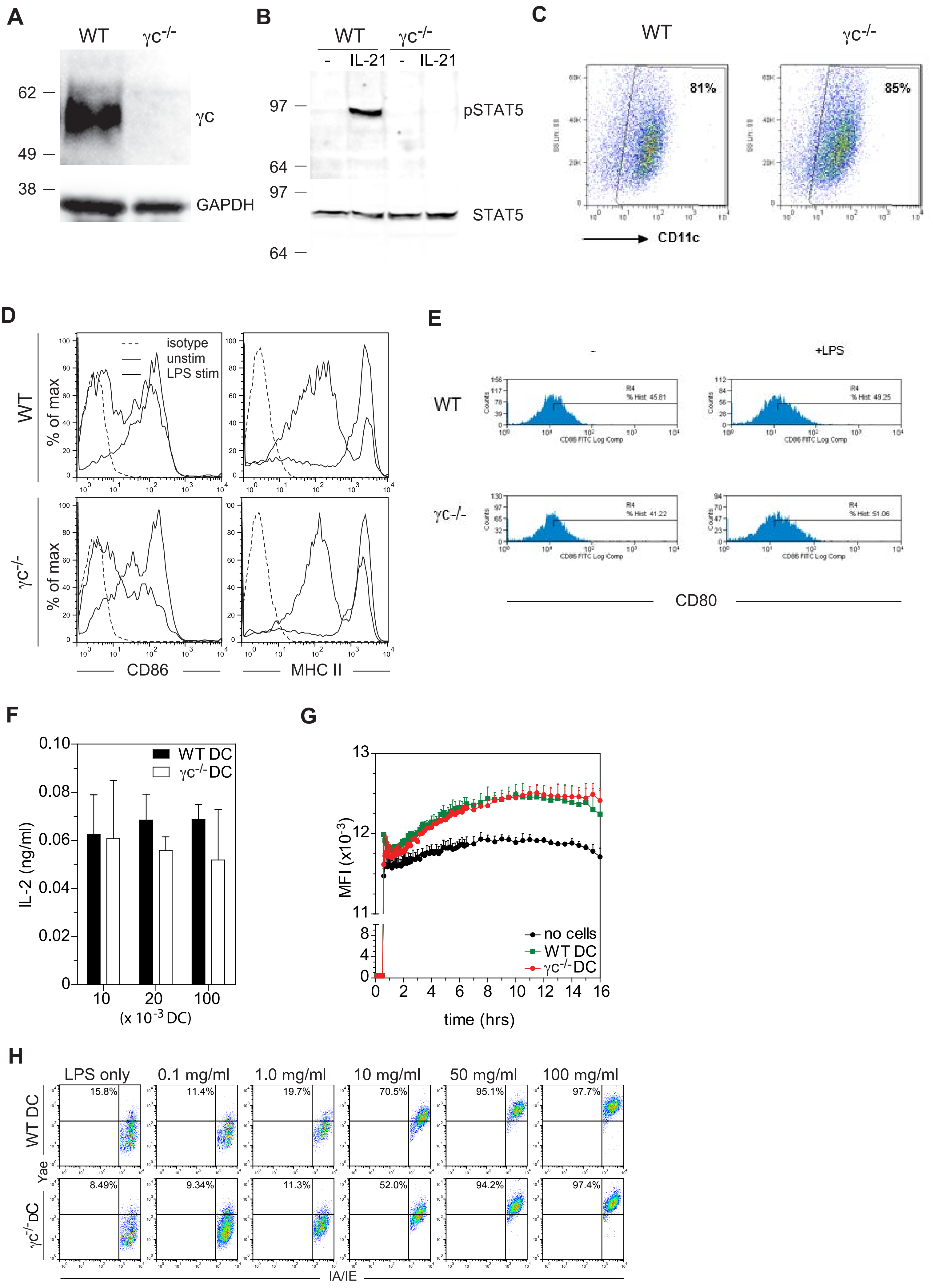
A
WT
γc-/-
62
49
38
γc
GAPDH
B
WT
γc-/-
-
IL-21
-
IL-21
97
64
97
64
pSTAT5
STAT5
C
WT
γc-/-
81%
85%
CD11c
D
isotype
unstim
LPS stim
WT
γc-/-
% of max
CD86
MHC II
E
-
+LPS
WT
γc-/-
CD80
F
WT DC
γc-/- DC
IL-2 (ng/ml)
0.10
0.08
0.06
0.04
0.02
0
10
20
100
(x 10-3 DC)
G
MFI (x10-3)
13
12
11
8
4
0
no cells
WT DC
γc-/- DC
0
2
4
6
8
10
12
14
16
time (hrs)
H
LPS only
0.1 mg/ml
1.0 mg/ml
10 mg/ml
50 mg/ml
100 mg/ml
WT DC
γc-/- DC
15.8%
11.4%
19.7%
70.5%
95.1%
97.7%
8.49%
9.34%
11.3%
52.0%
94.2%
97.4%
Yae
IA/IE

Fig. S2

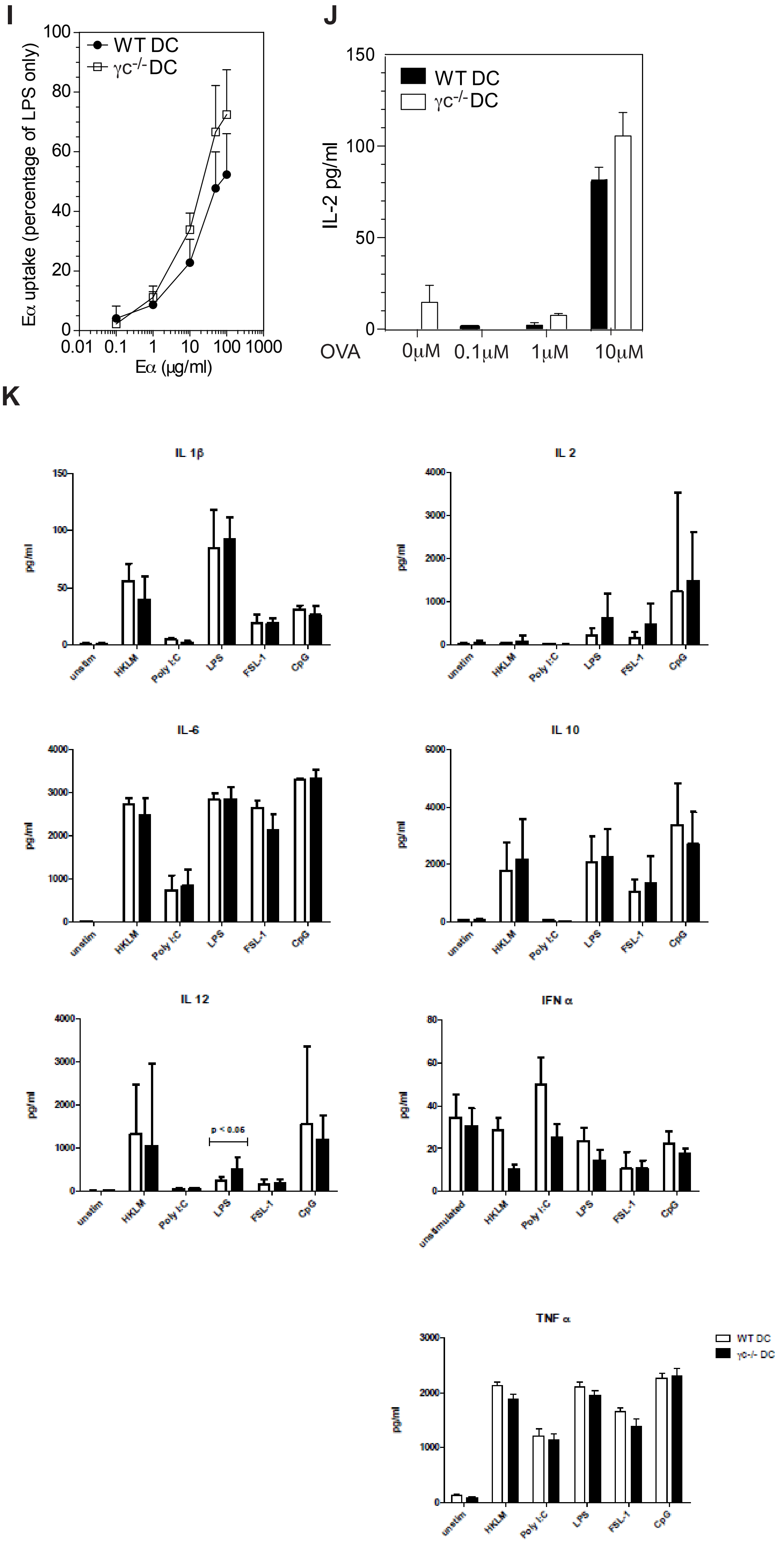
I
J
K
Eα uptake (percentage of LPS only)
WT DC
γc-/- DC
Eα (μg/ml)
0.01 0.1 1 10 100 1000
IL-2 pg/ml
OVA 0μM 0.1μM 1μM 10μM
IL 1β
IL 2
IL-6
IL 10
IL 12
IFN α
TNF α
pg/ml
p < 0.05
unstim
unstimulated
HKLM
Poly I:C
LPS
FSL-1
CpG
WT DC
γc-/- DC

Fig. S3

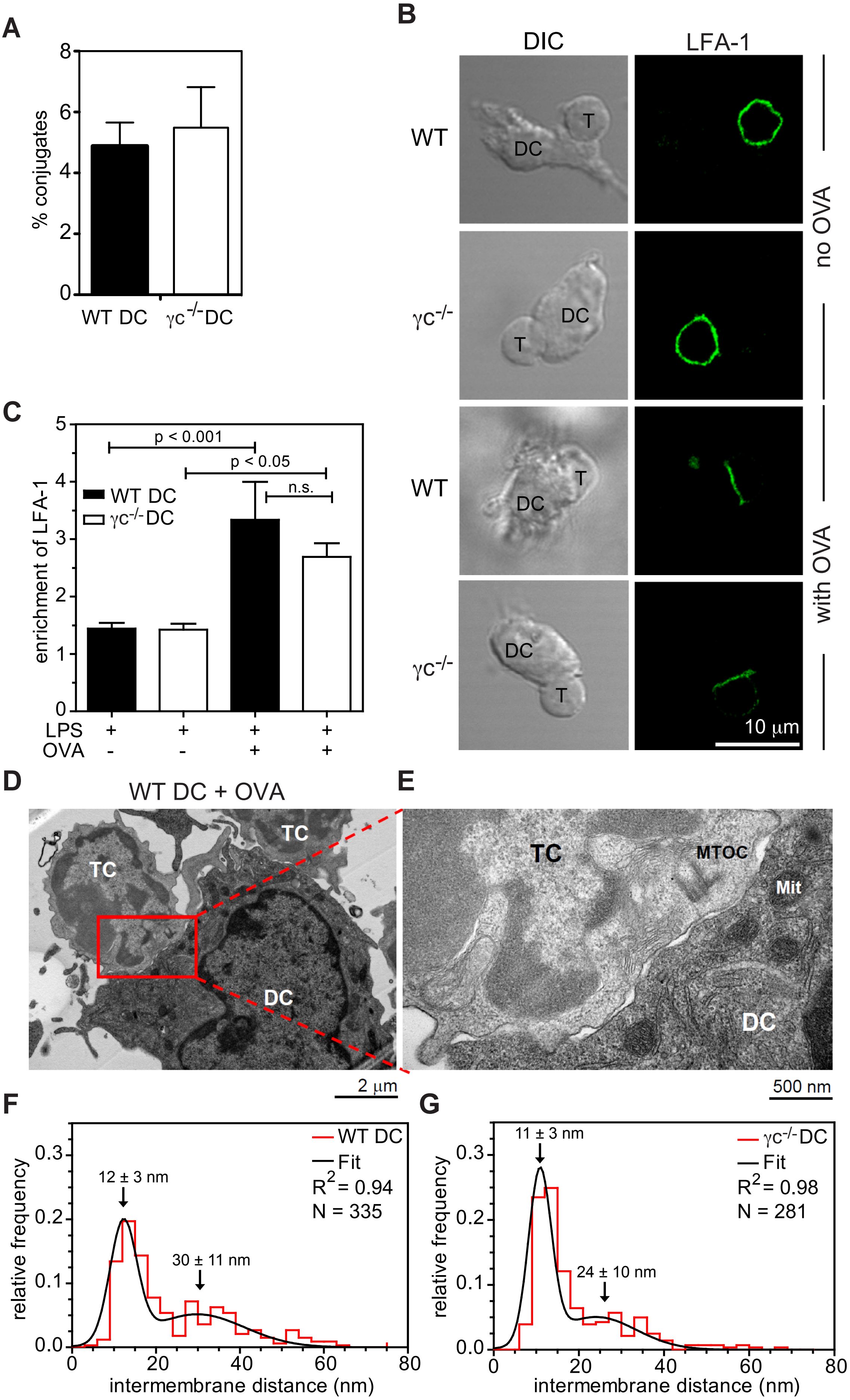
A
% conjugates
8
6
4
2
0
WT DC
γc-/-DC
B
DIC
LFA-1
WT
γc-/-
WT
γc-/-
DC
T
no OVA
with OVA
10 μm
C
enrichment of LFA-1
5
4
3
2
1
0
p < 0.001
p < 0.05
n.s.
WT DC
γc-/-DC
LPS + + + +
OVA - - + +
D
WT DC + OVA
TC
DC
2 μm
E
TC
MTOC
Mit
DC
500 nm
F
relative frequency
0.3
0.2
0.1
0.0
12 ± 3 nm
30 ± 11 nm
WT DC
Fit
R2= 0.94
N = 335
0
20
40
60
80
intermembrane distance (nm)
G
relative frequency
11 ± 3 nm
24 ± 10 nm
γc-/-DC
Fit
R2= 0.98
N = 281
intermembrane distance (nm)

Fig. S4

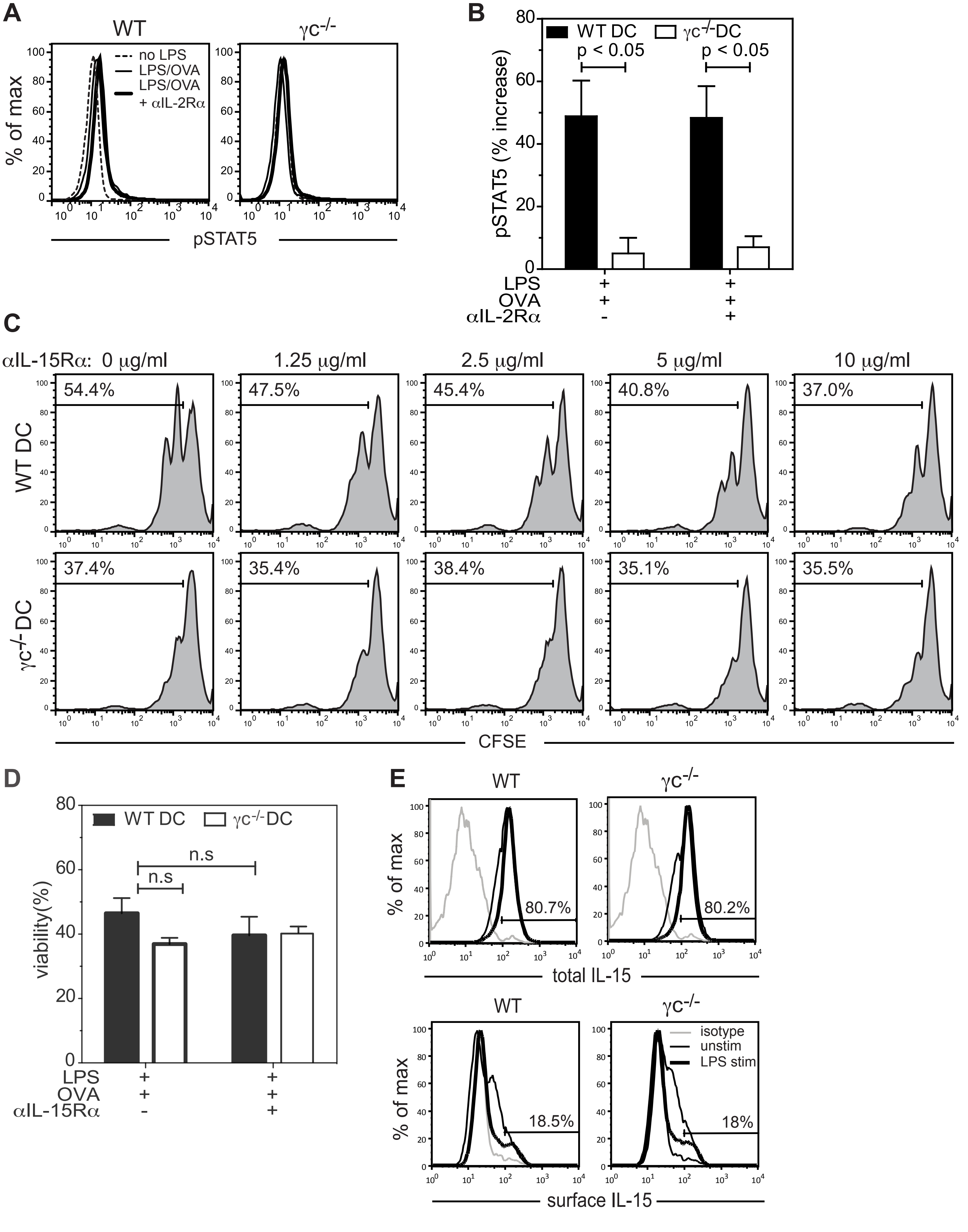

A
WT
γc-/-
no LPS
LPS/OVA
LPS/OVA + αIL-2Rα
% of max
pSTAT5
B
WT DC
γc-/-DC
p < 0.05
p < 0.05
pSTAT5 (% increase)
LPS
OVA
αIL-2Rα
C
αIL-15Rα: 0 μg/ml
1.25 μg/ml
2.5 μg/ml
5 μg/ml
10 μg/ml
WT DC
γc-/- DC
54.4%
47.5%
45.4%
40.8%
37.0%
37.4%
35.4%
38.4%
35.1%
35.5%
CFSE
D
WT DC
γc-/-DC
n.s
n.s
viability(%)
LPS
OVA
αIL-15Rα
E
WT
γc-/-
% of max
80.7%
80.2%
total IL-15
isotype
unstim
LPS stim
18.5%
18%
surface IL-15

Fig. S4

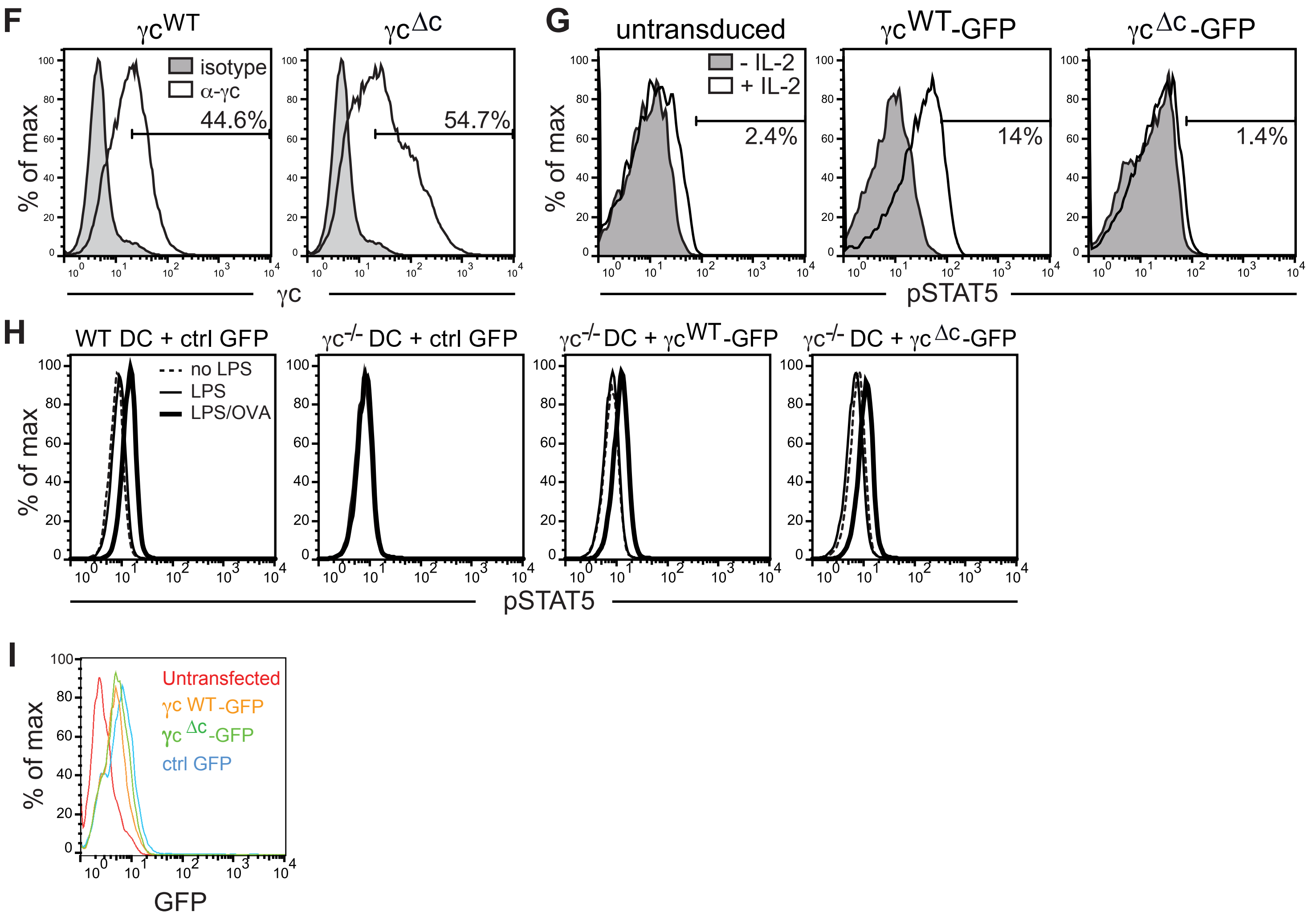
F
γc^WT
γc^Δc
isotype
α-γc
44.6%
54.7%
% of max
γc
G
untransduced
γc^WT-GFP
γc^Δc-GFP
- IL-2
+ IL-2
2.4%
14%
1.4%
% of max
pSTAT5
H
WT DC + ctrl GFP
γc-/- DC + ctrl GFP
γc-/- DC + γc^WT-GFP
γc-/- DC + γc^Δc-GFP
no LPS
LPS
LPS/OVA
% of max
pSTAT5
I
Untransfected
γc WT-GFP
γc Δc-GFP
ctrl GFP
% of max
GFP

Fig. S5

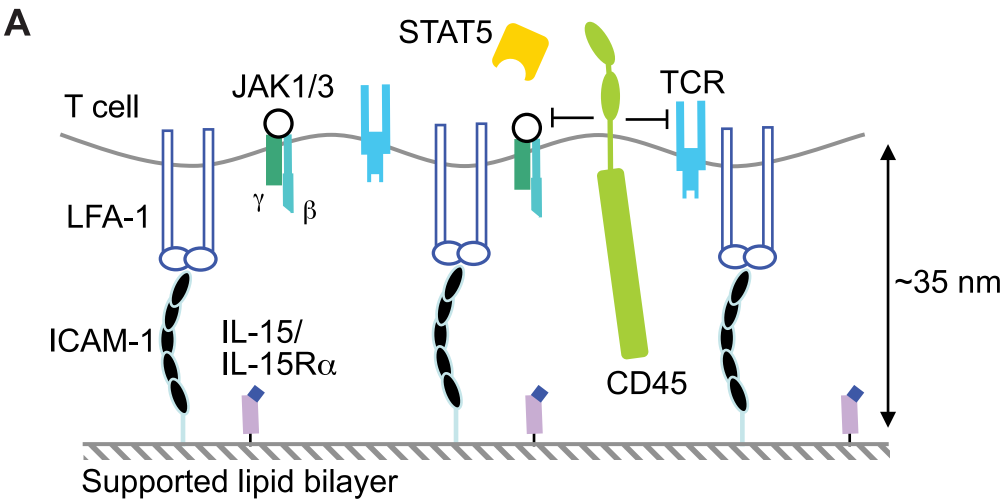

A
STAT5
JAK1/3
TCR
T cell
γ
β
LFA-1
~35 nm
ICAM-1
IL-15/
IL-15Rα
CD45
Supported lipid bilayer

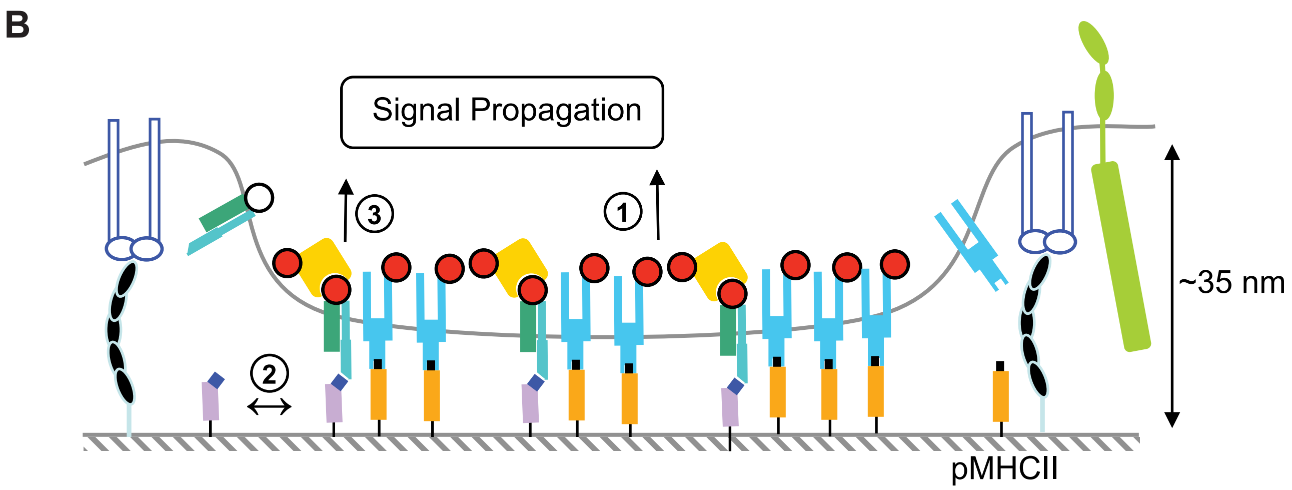

B
Signal Propagation
3
1
2
~35 nm
pMHCII

Fig. S6

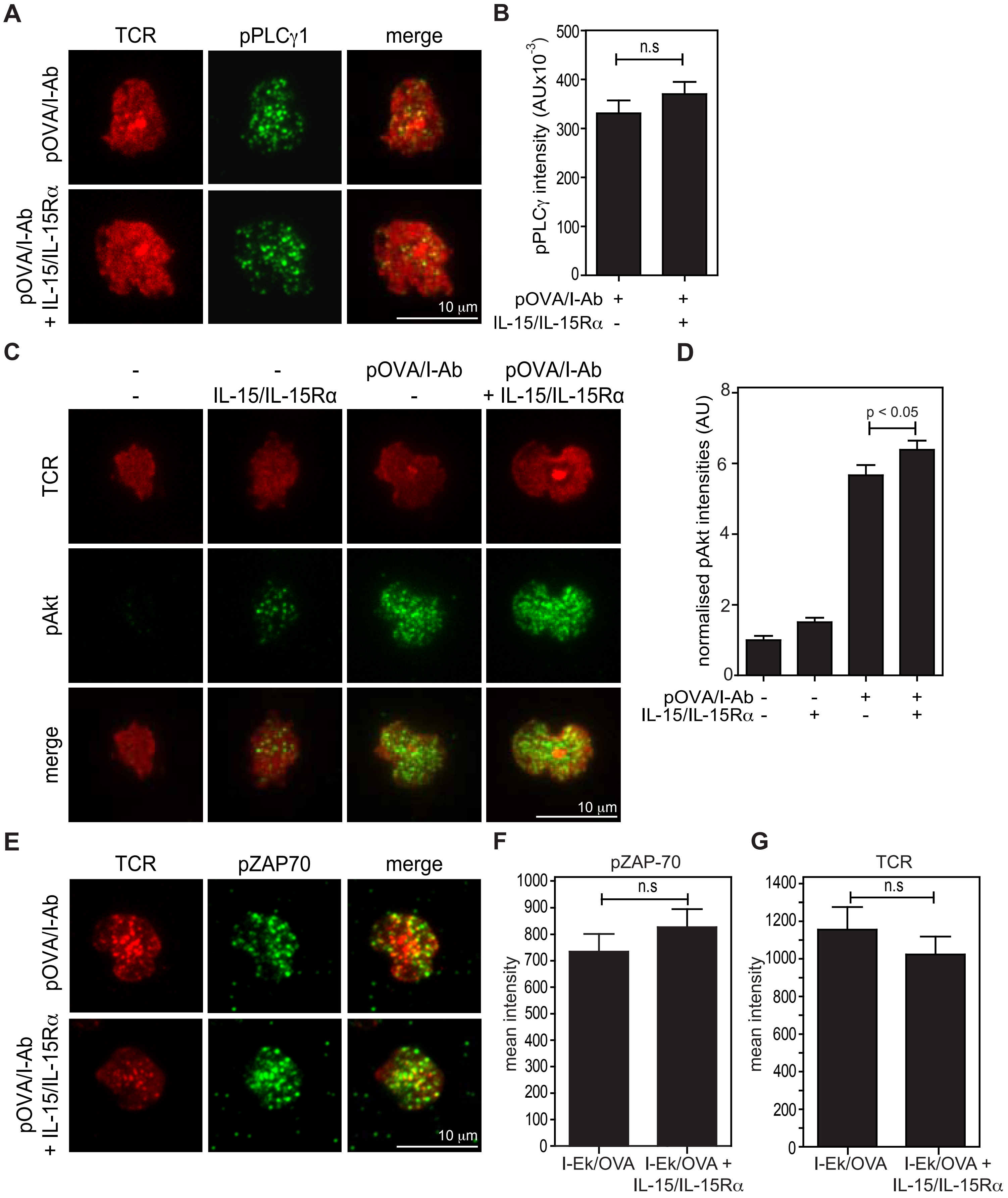

A
TCR
pPLCγ1
merge
pOVA/I-Ab
pOVA/I-Ab + IL-15/IL-15Rα
10 μm
B
pPLCγ intensity (AUx10^-3)
n.s
pOVA/I-Ab + +
IL-15/IL-15Rα - +
C
- -
- IL-15/IL-15Rα
pOVA/I-Ab -
pOVA/I-Ab + IL-15/IL-15Rα
TCR
pAkt
merge
10 μm
D
normalised pAkt intensities (AU)
p < 0.05
pOVA/I-Ab - - + +
IL-15/IL-15Rα - + - +
E
TCR
pZAP70
merge
pOVA/I-Ab
pOVA/I-Ab + IL-15/IL-15Rα
10 μm
F
pZAP-70
n.s
mean intensity
I-Ek/OVA
I-Ek/OVA + IL-15/IL-15Rα
G
TCR
n.s
mean intensity
I-Ek/OVA
I-Ek/OVA + IL-15/IL-15Rα

Fig. S7

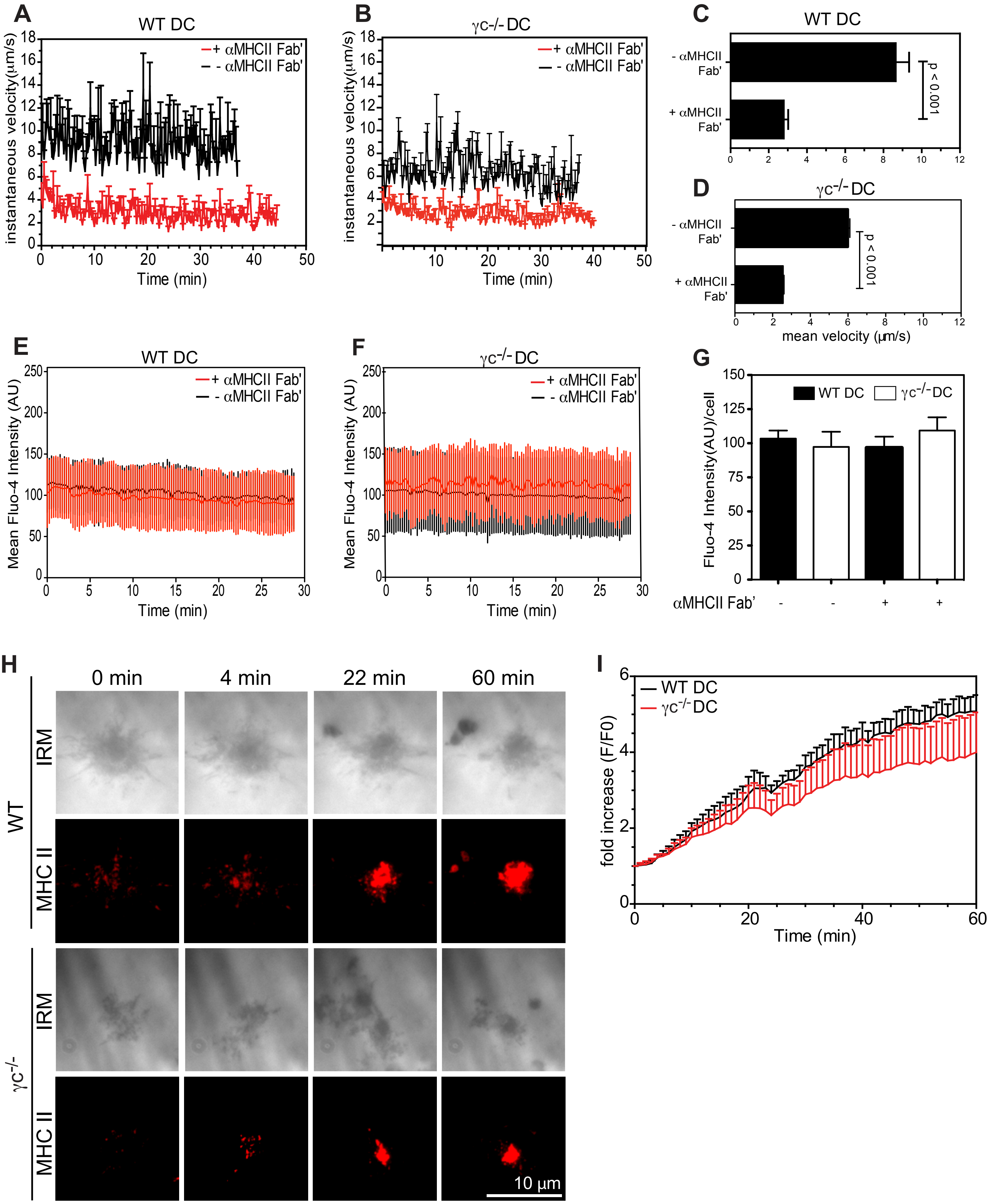
A
WT DC
+ αMHCII Fab'
- αMHCII Fab'
instantaneous velocity(μm/s)
Time (min)
B
γc-/- DC
+ αMHCII Fab'
- αMHCII Fab'
instantaneous velocity(μm/s)
Time (min)
C
WT DC
- αMHCII Fab'
+ αMHCII Fab'
p < 0.001
D
γc-/- DC
- αMHCII Fab'
+ αMHCII Fab'
p < 0.001
mean velocity (μm/s)
E
WT DC
+ αMHCII Fab'
- αMHCII Fab'
Mean Fluo-4 Intensity (AU)
Time (min)
F
γc-/- DC
+ αMHCII Fab'
- αMHCII Fab'
Mean Fluo-4 Intensity (AU)
Time (min)
G
WT DC
γc-/- DC
Fluo-4 Intensity(AU)/cell
αMHCII Fab' - - + +
H
0 min
4 min
22 min
60 min
WT
IRM
MHC II
γc-/-
IRM
MHC II
10 μm
I
WT DC
γc-/- DC
fold increase (F/F0)
Time (min)

Fig. S8

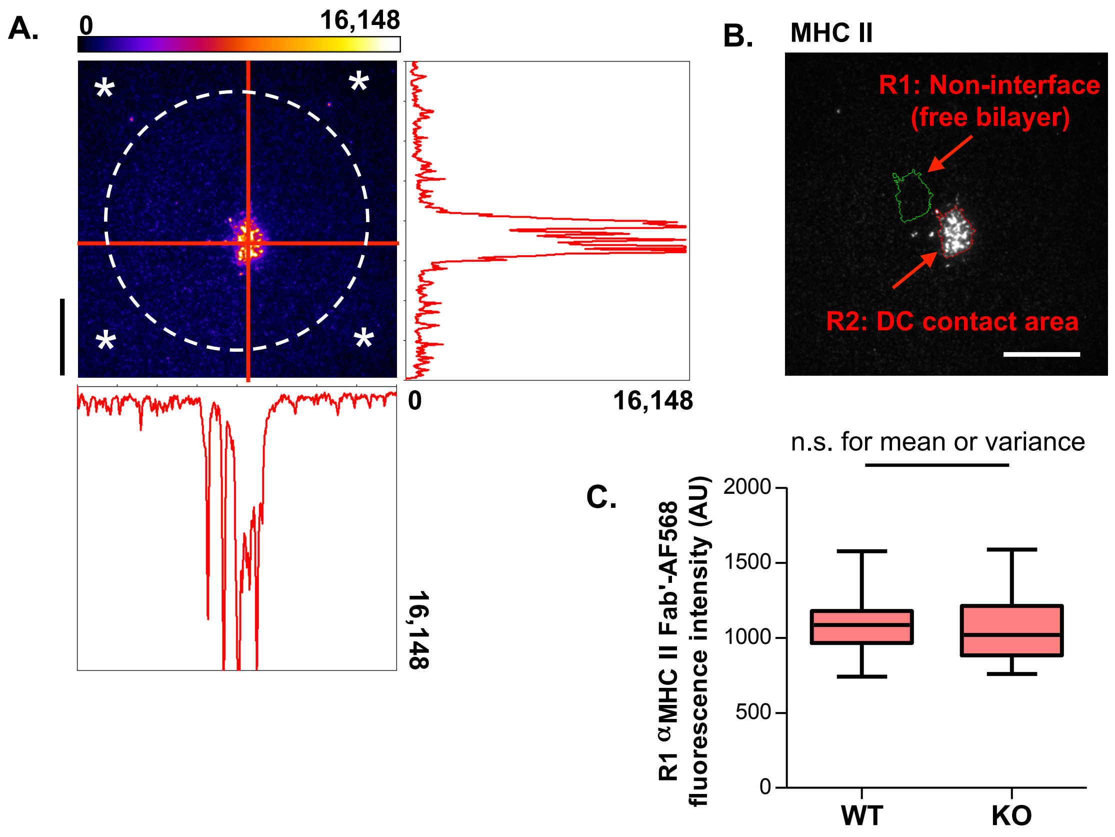
A.
0
16,148
0
16,148
16,148
B.
MHC II
R1: Non-interface (free bilayer)
R2: DC contact area
C.
n.s. for mean or variance
R1 αMHC II Fab'-AF568 fluorescence intensity (AU)
2000
1500
1000
500
0
WT
KO

Fig. S9

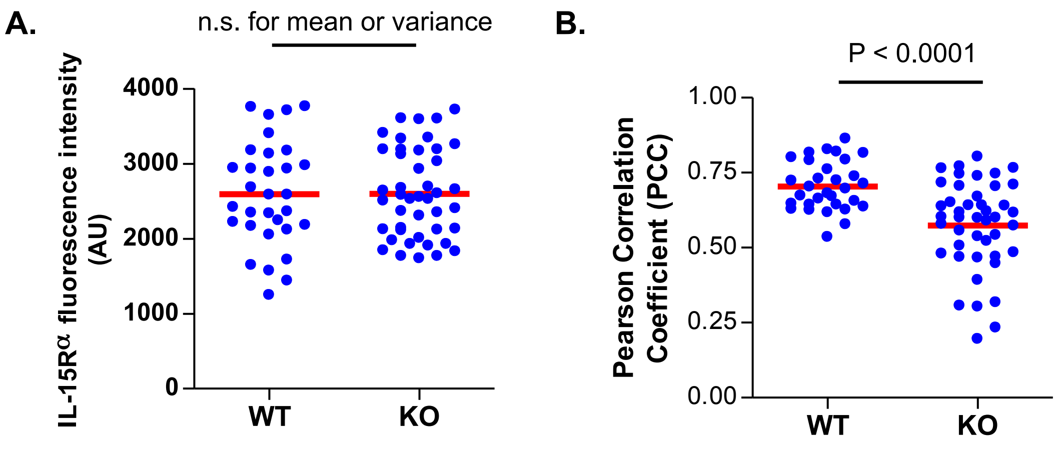
A.
n.s. for mean or variance
IL-15Rα fluorescence intensity (AU)
4000
3000
2000
1000
0
WT
KO
B.
P < 0.0001
Pearson Correlation Coefficient (PCC)
1.00
0.75
0.50
0.25
0.00
WT
KO
