# Supplementary material for: Dendritic cell-expressed common gamma-chain recruits IL-15 for trans-presentation at the murine immunological synapse
Source: Wellcome Open Res. 2018 Oct 17;3:84. Originally published 2018 Jul 17. [Version 2] doi: 10.12688/wellcomeopenres.14493.2 (PMC6234741; doi:10.12688/wellcomeopenres.14493.2)
Supplement: Supplementary file 2 [file wellcomeopenres-3-16191-s0001.tgz › 2e35fac5-76aa-494d-bab0-c7b316927fdb_Supplementary_file_2.zip!/Supplemental_figures_information_revised.docx.docx]

**Supplementary File 2: Supplemental figures.**

**Figure S1. yc deficiency alters the number but not the phenotype of splenic DC phenotype**

Single cell suspensions were obtained from the spleens of WT and γc/Rag 2-/- C57BL/6 mice and enriched for CD11c+ DCs by magnetic bead selection. (A) CD11c+ DC frequency per spleen (B) Density plot of DCs (gated on CD11c+ cells) stained with anti-CD11b, CD8α and B220 to identify CD11c+ CD11b+ CD8α-, CD11c+ CD8α+ CD11b- and CD11c+ B220+ cells. (C) Mean percentage of CD11c+ CD11b+, CD11c+ CD8α+ and CD11c+ B220+ within total CD11c+ cells in spleens of 3 WT and γc/Rag 2 -/-. P values, two-tailed students t-test.

**Figure S2. Absence of γc signaling does not affect DC maturation in response to activation stimuli or antigen handling.** (A) Immunoblot analysis of γc expression in WT and γc^-/-^ DC. GAPDH serves as a loading control. (B) pSTAT5 expression in WT and γc^-/-^ DC cultured for 10 min with/without IL-21 (100 ng/ml). STAT5 serves to demonstrate equal total STAT5 levels. (C) Surface expression of CD11c. (D) CD86 and MHCII expression in DC cultured overnight with or without LPS. (E) CD80 expression in DC cultured overnight with or without LPS (F) IL-2 release by DC stimulated with LPS and OVA for 72hrs. (G) Uptake and breakdown of DQ-OVA by LPS-matured DC, measured as emission of green fluorescence (515nm). (H) Representative plots showing Eα peptide presentation in CD11c^+^IA/IE^hi^ gated cells. (I) Quantification of Eα peptide uptake (H), shown as an index relative to Yae expression of LPS-matured DC. (J) IL-2 production by BO17.4 hybridoma cells. (K) Cytokine release by CD11c-selected DC after overnight maturation with LPS (100ng/ml). Data are from 3 (G-J) or 8 independent experiments (K) (mean ± S.E.M). *P* values, Student’s *t*-test.

**Figure S3. γc deficiency does not affect T cell adhesion to DC.** (A) Quantification of conjugate formation between DDAO-labelled CD4+ T cells and CFMDA-labelled DC activated with LPS and pulsed with OVA (1:1 ratio). DC were brought into contact with T cells by centrifugation. After 1 hr incubation, cells were analysed by flow-cytometry. Data are from 3 independent experiments (mean ± S.E.M) (all differences are n.s). (B) Representative confocal images of T-DC conjugates showing localization of LFA-1 (green). (C) Quantification of T cell LFA-1 enrichment at the IS (B), expressed as ratio of fluorescence intensity at the contact interface versus non-contact perimeter of T cells. Data are from 2 independent experiments (N=35–48, mean ± S.E.M). *P* values, one-way ANOVA. (D) Representative transmission electron micrograph of CD4+ T cells (TC) interacting with WT DC (magnification 8,800x). (E) High magnification image of the region outlined in red box in D (magnification 19,500x). MTOC, microtubule organising center; Mit, mitochondrion. (F-G) Frequency distribution (red lines) of intermembrane distances measured at WT (N=12)(F) and γc^-/-^ DC (N=9) (G) conjugates with CD4+ T cells at regions of parallel membrane apposition along the contact interface. Black lines indicate fits of the distributions to a two-Gaussian distribute. Fits with a single Gaussian model were inferior, R^2^ 0.71 and 0.88 for WT and γc^-/-^ DC respectively, while three-Gaussian models did not converge. Optical imaging was performed using a Zeiss LSM 510 confocal microscope controlled by Zen software, using a x63 oil objective, N.A. 1.4. Electron microscopy was performed using a Phillips CM12 transmission electron microscope equipped with a 4k x 2.7k digital camera (Gatan).

**Figure S4. DC-expressed γc is required for transpresentation of IL-15 to CD4+ T cells.** (A) pSTAT5 induction in CD4+ T cells following incubation with DC, either untreated or stimulated overnight with LPS +OVA. DC were blocked with anti-IL-2Rα or isotype control. (B) Quantification of increase in pSTAT5 fluorescence (A), compared to unstimulated control. Data are from 3 independent experiments (mean ± S.E.M). *P* values, Student’s *t*-test. (C) Representative plots showing CD4+ T cell proliferation, measured by CFSE dilution, after 72hr co-culture with OVA-pulsed DC pre-treated with anti-IL-15Rα at the indicated concentrations (DC:T cell ratio 1:5). Gates are set on T cells cultured with DC without antigen. (D) Quantification of cell viability, as measured by AnnexinV-PI staining, in CD4+ T cells after co-culture with LPS-matured OVA-pulsed DC for 72 hrs. DC were blocked with anti-IL-15Rα or isotype control. Data are from 3 independent experiments (mean ± S.E.M). *P* values, Student’s *t*-test. (E) Total and surface IL-15 expression on CD11c gated cells. % are indicated for LPS-stimulated cells. Plots are representative of 3 independent experiments. (F-G) Human ED-7R cells were infected with lentiviral vectors encoding γc^WT^GFP and γc^Δc^-GFP. After 72 hours, cells were stained with anti-γc antibody (F) or stimulated with/out IL-2 (100ng/ml) and subsequently stained with anti-pSTAT5 antibody (G). (H) pSTAT5 induction in CD4+ T cells following incubation with DC, transfected with γc^WT^-GFP, γc^Δc^-GFP or ctrl GFP, then stimulated as indicated. (I) Levels of GFP expression obtained by nucleofecting DCs with γc^WT^-GFP, γc^Δc^-GFP or ctrl GFP.

**Figure S5. Model for potentiation of IL-15 mediated trans-signaling in CD4+ T cells by TCR engagement.** (A-B) Mass action-mediated trapping of IL-15Rα/IL-15 complexes at the T cell IS with planar lipid bilayers. (A) The long span of ICAM-1/LFA-1 complexes, in the absence of TCR engagement, would be expected to position the T cell plasma membrane at a distance that is incompatible with efficient IL-15Rα/IL-15 binding to T cell surface IL-2Rβγ heterodimers. (B) Engagement of TCR by planar bilayer-tethered pMHCII would permit freely diffusing planar bilayer-tethered IL-15Rα/IL-15 complexes to bind and trigger IL-2Rβγ heterodimers at regions of close contact. This model implies that TCR engagement is permissive for STAT5 activation by IL-15Rα/IL-15 at the T cell IS. The binding efficiency of IL-15Rα/IL-15 complexes by IL-2Rβγ heterodimers at sites of TCR engagement in T cell-DC contacts may be more efficient, due to spatial coupling of IL-15Rα/IL-15 to pMHC II by γc.

**Figure S6. IL-15 trans-signaling does not contribute to TCR signaling.** CD4+ T cells were incubated on glass-supported planar bilayers containing ICAM-1, CD80 and pOVA/I-A^b^ or IL-15/IL15Rα-6his or both as indicated. After 30min incubation at 37°C, cells were fixed, permeabilized, and stained for TCR (red for all) and pPLCγ1 (green)(A,B), pAKT (green)(C,D), pZAP70 (green)(E,F) or TCR (G). Representative TIRFM images (A,C,E) and quantification in arbitrary units (AU) (B,D,F-G) of mean fluorescence intensities at contact interfaces (N=35-108, mean ± S.E.M). *P* values, one-way ANOVA. Imaging was performed on a Nikon Ti microscope with a 100x TIRF objective, N.A. 1.49, controlled by Nikon Elements software. Fluorescence images were captured using an Ixon cooled EMCCD camera (512 x 512 pixels, Andor Technology). Mean fluorescence intensity at contact interfaces was quantified from 14 bit images using Metamorph software.

**Figure S7. Ligation of MHCII results in DC motility arrest and in MHC II accumulation at the contact interface.** LPS/OVA stimulated DC in HBS/HSA buffer were incubated on glass-supported planar bilayers containing LFA-1α1 ± I-A/E Fab’. (A,B) Instantaneous velocity over time and (C,D) mean velocity of DC incubated on bilayers for 37-45 min (N=16-28, mean ± S.E.M). (E-F) Time course of Fluo-4 intensity and (G) mean Fluo-4 intensity in arbitrary units (AU) at the contact interface of DC incubated on bilayers for 30 min (N=15-20, mean ± S.E.M). (H) Representative TIRFM images demonstrating MHC II (red, Alexa Fluor 568) accumulation at contact interfaces of WT (N=6) and γc^-/-^ (N=10) DC incubated on bilayers containing LFA-1 α1 + I-A/E Fab’ (Alexa Fluor 568) for 60 min. Corresponding interference reflection microscopy (IRM) images delineate the contact area. (I) Time course of total bound MHCII fluorescence at contact interfaces (F), shown in H, normalized relative to fluorescence at time of initial contact t=0 (F_0_), expressed as fold increase (F/F_0_), (mean + S.E.M. is shown for clarity). Cell tracking and intracellular Ca^2+^ imaging was performed using a Zeiss LSM510 confocal microscope, and a 20x air objective N.A. 0.8. MHC II clustering was followed using a Nikon Ti microscope with a 100x TIRF objective, N.A. 1.49, and fluorescence captured using an Ixon cooled EMCCD camera (512 x 512 pixels, Andor Technology). Cell tracking was performed using ImageJ software. Fluorescence intensity was quantified using Metamorph software.

**Figure S8. TIRF field illumination is comparable between WT and γc^-/-^ DC samples. (**A) Representative 14-bit 512 x 512 image of a TIRF field of bilayers containing αMHC II Fab’-AF568 (and unlabeled LFA-1 I-domain) with a centrally positioned WT DC contact interface. The image is presented as a pseudocolor heatmap to more clearly depict the non-interface αMHC II Fab’-AF568 fluorescence (blue). Linescans represent fluorescence intensity across the field with the DC contact interface at the center. Asterisks indicate areas of maximum drop-off in TIRF-illumination at the extreme edges of the field, due to optical aberration and beam morphology. Dashed circle represents a central region of the filed with more even illumination. (B) The same image as in (A*)* depicted in grayscale, showing non-interface (R1) and the DC contact interface region (R2), from which fluorescence intensity was measured. (C) Mean and inter-quartile range of αMHC II Fab’-AF568 fluorescence intensity for all non-interface regions adjacent to interfaces from which IL-15Rα fluorescence intensity was measured. Bars represent S.D., line boxes represent mean and interquartile interval. Mean and variance were compared with Student *t*-test and *F*-test respectively. Scale bars, 20 μm. Imaging was performed on a Nikon Ti microscope with a 100x TIRF objective, N.A. 1.49, controlled by Nikon Elements software.

**Figure S9. At DC interfaces with comparable IL-15R**α **levels, engaged MHC II remains less colocalized with IL-15R**α **in the absence of** γ**c.** Values for IL-15Rα fluorescence intensity in arbitrary units (AU) at DC interfaces with bilayers containing αMHC II Fab’-AF568 and LFA-1 I-domain were numerically ordered for WT and γc^-/-^ cells. (A) Contiguous values in each group were selected (to preserve variance), from the overlap range between WT (n = 31) and γc^-/-^ DC (n = 44) interfaces, for calculation of Pearson’s correlation coefficient (PCC). The mean and variance of IL-15Rα fluorescence intensity at interfaces in the two groups were compared using Student *t*-test and *F*-test respectively. (B) PCC was calculated for all contact interfaces in (A) using IL-15Rα and MHC II fluorescence channels. Means were compared by Student *t*-test. Dots represent individual cell interfaces, and red bars represent sample means. Imaging was performed on a Nikon Ti microscope with a 100x TIRF objective, N.A. 1.49, controlled by Nikon Elements software.
