# Supplementary material for: Dendritic cell-expressed common gamma-chain recruits IL-15 for trans-presentation at the murine immunological synapse
Source: Wellcome Open Res. 2018 Oct 17;3:84. Originally published 2018 Jul 17. [Version 2] doi: 10.12688/wellcomeopenres.14493.2 (PMC6234741; doi:10.12688/wellcomeopenres.14493.2)
Supplement: Supplementary file 3 [file wellcomeopenres-3-16191-s0002.tgz › 6d78f066-bc09-45f2-94ef-426384d2c820.zip!/Supplementary File 3/Supplemental movie information.docx]

**Supplementary File 3: Supplemental movies.**

**Movie S1. WT DC exhibit ‘crawling’ motility on an LFA-1 α1 planar bilayer surface.** LPS/OVA-stimulated WT DC were incubated on bilayers containing LFA-1 α1 and imaged by confocal microscopy at 37°C for 37 min. DIC, differential interference contrast channel, IRM, reflection channel using 488 nm laser illumination, Tracks, colored cell tracks acquired manually using DIC images (matching spots represents initial position of cells).

**Movie S2. WT DC rapidly undergo motility arrest and accumulate MHCII at the contact interface following MHCII ligation.** LPS/OVA-stimulated WT DC were incubated on bilayers containing LFA-1 α1 and IA/E Fab’-AF568, and imaged by confocal microscopy at 37°C for 45 min. DIC, differential interference contrast channel, IRM, reflection channel using 488 nm laser illumination, Tracks, colored cell tracks acquired manually using DIC images (matching spots represents initial position of cells). Loss of fluorescence intensity over time in the AF568 channel is due to a combination of focal drift and photobleaching.

**Movie S3. WT and γc^-/-^ DC undergo comparable motility arrest following MHC ligation.** Panels show representative DIC image timeseries acquired at 15 s intervals of WT and γc^-/-^ DC interacting with bilayers containing LFA-1 α1, with or without IA/E Fab’-AF568. Dots represents cell centroid, and lines represent translation/frame.

**Movie S4. MHC ligation does not increase intracellular Ca^2+^ in WT DC.** Panels show representative Fluo-4 loaded WT DC imaged at 15s intervals interacting with planar bilayers containing LFA-1 α1, with or without IA/E Fab’-AF568.

**Movie S5. MHC ligation does not increase intracellular Ca^2+^ in γc^-/-^ DC.** Panels show representative Fluo-4 loaded γc^-/-^ DC imaged at 15s intervals interacting with planar bilayers containing LFA-1 α1, with or without IA/E Fab’-AF568.

**Movie S6. MHCII ligation at the DC contact interface results in MHCII clustering and central transport.** LPS/OVA-stimulated DC were transfected with ctrl GFP (to delineate the contact area), and imaged on planar bilayers containing LFA-1 α1 and IA/E Fab’-AF568 by TIRFM at 37°C for 27 min. Representative TIRFM real-time imaging of GFP (green) and MHCII accumulation (red) at the contact DC contact interface with the supported bilayer. Merge, overlay of GFP and MHCII channels.

**Movie S7. Binding-induced MHC clustering and transport in WT and γc^-/-^ DC. a.** Following MHC ligation, discrete MHCII microclusters form throughout the contact interface in WT and γc^-/-^ DC, which rapidly organize into multifocal intermediate clusters (mesoclusters), that are transported to the IS center. Mesoclusters are not stable entities, and are prone to fragmentation and coalescence throughout their route to the contact center. At least two other modes of MHCII transport occur in both WT and γc^-/-^ DC. **b.** Sequential transport of MHCII microclusters along filopodial extensions. **c.** Episodic lamelopodial extensions nucleate MHCII microclusters (seen within yellow boxed region) and are ‘swept’ into the central contact area, where they join MHCII mesoclusters for central transport. **Right panels** show timeseries thresholded for MHCII intermediate clusters, to illustrate there accumulation and transport towards the contact center. Contrast and brightness were adjusted equally for WT and γc^-/-^ DC image series to more clearly delineate MHCII clusters.
