# Supplementary material for: Dendritic cell-expressed common gamma-chain recruits IL-15 for trans-presentation at the murine immunological synapse
Source: Wellcome Open Res. 2018 Oct 17;3:84. Originally published 2018 Jul 17. [Version 2] doi: 10.12688/wellcomeopenres.14493.2 (PMC6234741; doi:10.12688/wellcomeopenres.14493.2)
Supplement: Peer review history

# Peer review: Referee response for version 2

**DOI:** 10.21956/wellcomeopenres.16191.r34086

**Published:** 2018-11-08

**Authors:** Matthew Collin

**Affiliations:** 1 Institute of Cellular Medicine, Newcastle University, Newcastle upon Tyne, UK; 2 NIHR Newcastle BRC, Newcastle Hospitals NHS Foundation Trust, Newcastle, UK

Thank you for addressing the comments.

I have read this submission. I believe that I have an appropriate level of expertise to confirm that it is of an acceptable scientific standard.

# Peer review: Referee response for version 2

**DOI:** 10.21956/wellcomeopenres.16191.r34087

**Published:** 2018-10-22

**Authors:** Jung-Hyun Park, Tae-Hyoun Kim

**Affiliations:** 1 Experimental Immunology Branch, Center for Cancer Research, National Cancer Institute, National Institutes of Health, Bethesda, MD, USA; 2 Experimental Immunology Branch, Center for Cancer Research, National Cancer Institute, NIH, Bethesda, MD, USA

We have no further comments to make.

We have read this submission. We believe that we have an appropriate level of expertise to confirm that it is of an acceptable scientific standard.

# Peer review: Referee response for version 1

**DOI:** 10.21956/wellcomeopenres.15778.r33724

**Published:** 2018-09-10

**Authors:** Jung-Hyun Park, Tae-Hyoun Kim

**Affiliations:** 1 Experimental Immunology Branch, Center for Cancer Research, National Cancer Institute, NIH, Bethesda, MD, USA; 2 Experimental Immunology Branch, Center for Cancer Research, National Cancer Institute, National Institutes of Health, Bethesda, MD, USA

The cytokine IL-15 employs a unique mechanism called “trans-presentation” for signaling. IL-15 transpresentation is distinct to conventional cytokine signaling mechanisms because it requires cell-cell contact between IL-15 expressing and IL-15 signaled cells, and because IL-15 needs to be complexed with its proprietary IL-15Ra-chain to be utilized by target cells. Dendritic cells are potent producers of IL-15. Dendritic cells also play a critical role in antigen presentation to T cells, but it has not been known whether there is crosstalk between these two events. Moreover, it has remained unclear whether these events need to be induced by the same cell or can be triggered by different dendritic cells. Understanding these issues is critical to gain further insights into the regulatory mechanisms of T cell immunity.

 The current study by Beilin and colleagues now reports a previously unappreciated requirement for cognate peptide-MHC-II/TCR engagement in IL-15 transpresentation by dendritic cells. Mechanistically, the authors report that the common gamma-chain (gc) cytokine receptor on dendritic cells facilitates IL-15 signaling by recruiting the IL15/IL-15Ra complex to the immunological synapse. Importantly, copatching of gc and IL-15/IL-15Ra was independent of gc receptor signaling, suggesting a recruitment mechanism that is presumably mediated by the receptor ectodomains. Collectively, these findings report a new layer of control in IL-15- transpresentation that directly impacts antigen-specific priming of CD4+ T cells and consequently the establishment of protective T cell immunity.

 This study is interesting in two ways:

 Firstly, it reveals a previously unappreciated role for gc proteins in IL-15 signaling, that is surprisingly required on IL-15-producing cells – and not on target cells.

 Secondly, it reveals a new requirement for TCR ligation to achieve effective IL-15 transpresentation, thus unveiling crosstalk between antigen-presentation and IL-15 transpresentation on the same dendritic cell.

 The observations are well documented, and the initial findings are nicely corroborated using a series of imaging studies where cell surface events were scaled down and assessed on lipid bilayers to minimize bystander events. I do not find additional experiments necessary, but I would consider it helpful if the following points could be addressed.

 A control experiment that can demonstrate the antigen specificity of the TCR/MHC-II engagement, which is proposed to be required for IL-15 transpresentation (Figure 1D, E), would be informative. Instead of using OT-II CD4 T cells, can the authors use wildtype CD4+ T cells and co-culture them with OVA preloaded DC? Based on the authors’ model, polyclonal wildtype CD4 T cells would fail to phosphorylate STAT5. Is this the case?

 Does the recruitment of IL-15/IL-15Ra by gc proteins depend on the cytokine IL-15? Would “empty” IL-15Ra proteins - that are not complexed with IL-15 - suffice to be recruited by gc into the immunological synapse? The authors have established a set of experimental tools in this study that could provide answers to these questions.

We have read this submission. We believe that we have an appropriate level of expertise to confirm that it is of an acceptable scientific standard.

# Response

**Published:** 2018-09-25

**Authors:** Siobhan Burns

**Affiliations:** Royal Free and University College, UK

Response to comment about antigen specificity:

Using co-cultures between OTII T-cell and wild type DC, we have shown the requirement of MHC-antigen loading for activation of T-cell proliferation and STAT5 phosphorylation (Figure 1 A, D and E) as well as for formation of the DC-T-cell immune synapse in that is a pre-requisite for IL-15 transpresentation in our model (Figure S3). We chose to further dissect whether antigen loading of MHCII is required for T-cell priming using our lipid bilayer model which has the advantage of reducing confounding variables to permit examination of specific elements of antigen presenting cell (APC):T-cell interaction. Data from this approach also supported the need for a cognate interaction between APC and T-cell for IL-15 transpresentation and STAT5 phosphorylation in T-cells (Figure 2A). We agree that future testing of the model we have proposed should include experiments using polyclonal T-cells as suggested to further address the issue of antigen specificity.

Response to comment about 'empty' IL-15Ra:

It was previously shown that IL-15 and IL-15Ra generated by DC form intracellular complexes prior to expression at the cell surface and that these proteins predominantly exist as a complex in DC (Mortier et al JEM 2008, reference 47).  Therefore, the degree and physiological relevance of ‘empty’ IL-15Ra at the immune synapse remains unclear.  As a structural question this is interesting as ligation increases the affinity of the interaction of most of the gc associated alpha chains by 1-2 logs (Gonnord P, Angermann BR, Sadtler K, Gombos E, Chappert P, Meier-Schellersheim M, Varma R. A hierarchy of affinities between cytokine receptors and the common gamma chain leads to pathway cross-talk. Sci Signal. 2018;11(524). doi: 10.1126/scisignal.aal1253. PubMed PMID: 29615515.). Response to point regardRe In these cases the unligated receptor interact with gc with a 10 ^-6^ M Kd , which may enable interaction in a synapse as its similar to the affinity of most TCR for agonist pMHC.  Therefore, we agree that this is an interesting avenue to explore going forward to test the relative importance of physical interaction and signalling for the model that we have proposed.

# Peer review: Referee response for version 1

**DOI:** 10.21956/wellcomeopenres.15778.r33507

**Published:** 2018-07-30

**Authors:** Matthew Collin

**Affiliations:** 1 Institute of Cellular Medicine, Newcastle University, Newcastle upon Tyne, UK; 2 NIHR Newcastle BRC, Newcastle Hospitals NHS Foundation Trust, Newcastle, UK

Beilin et al describe in vitro experiments in the murine system that implicate a role for trans-presentation of IL-15 in the activation of CD4+ T cells by GM-CSF stimulated BM-derived dendritic cells.  The clinical premise of the work is the observation that γc and JAK3 SCID patients are not protected from HPV infection after transplantation. The authors surmise that this is due to incomplete chimerism in the myeloid compartment although there are other possibilities as suggested by Reference 2. Laffort et al that uncorrected NK or epithelial cell function could be responsible.  The clinical inference made by the authors is tenuous since there are no data on Langerhans cell or dendritic cell chimerism in this patient group as far as I am aware. However, their argument does not detract from the conclusion that IL-15 trans-presentation is shown to be a key player in the immune synapse between MHC II and CD4 T cells.  The authors show that γc-deficient DC do not have obvious defects of MHCII expression, maturation, antigen uptake and processing, and adhesion to T cells (sup data), yet they are inferior in T cell activation assays (Figure 1).  This defect is at least partly reproduced by antibody blockade of IL15R in wild-type cells. The authors go on to dissect the mechanism of IL-15 trans-presentation showing that T cell STAT5 is only activated in the context of TCR ligation (Figure 2) that γc and IL-15R are recruited to the synapse (Figure 3,4,5) and that signalling to the DC is not required, through the use of a γc C-terminal truncation (Figure 5).  

 Points requiring clarification

 1) The GM-CSF stimulated BM DC has recently come under intense scrutiny as a heterogenous preparation of DC-lineage and monocyte-lineage cells (e.g Helft, J ^1^). The explanation that IL15 trans-presentation is the major defect in γc -/- preparations critically rests on these preparations being functionally equivalent (in all other respects) to WT cells. Given that GM-CSF also signals through STAT5, it is important to exclude an interaction between GM-CSF and γc in the generation of -/- DC.  For example, γc cytokines originating from a plethora of cells in the BM could enhance the function of wild-type preparations in some way. The authors could improve their description of BM-derived DC by presenting more detailed flow cytometry or bulk gene expression analysis of wild-type and  γc -/- preparations. The methods section should at least contain an outline of how the cells were obtained (the use of GM-CSF was gleaned from the results sections).

 2) In Figure 1, it would be useful to have more clarity concerning how much the defect in antigen presentation by γc-/- DC was attributable to IL-15, e.g in panel F the γc-/- are normalised to their own poor performance. Why not show the raw data rather than % inhibition? Related to this point, the authors cite a notable previous study in which trans-presentation of IL-2 was demonstrated to be essential at the immune synapse (14. Wuest et al). It was not clear from the data or interpretation what the relative importance of IL15 and IL2 trans-presentation would be since both could contribute to the inferior performance observed in the γc -/- phenotype.  This point could receive more attention in the discussion.

I have read this submission. I believe that I have an appropriate level of expertise to confirm that it is of an acceptable scientific standard, however I have significant reservations, as outlined above.

## References

[1] HelftJBöttcherJChakravartyPZelenaySHuotariJSchramlBUGoubauDReis e SousaC: GM-CSF Mouse Bone Marrow Cultures Comprise a Heterogeneous Population of CD11c(+)MHCII(+) Macrophages and Dendritic Cells. *Immunity*.2015;42(6) : 10.1016/j.immuni.2015.05.018 1197-211 10.1016/j.immuni.2015.05.018 26084029

# Response

**Published:** 2018-09-25

**Authors:** Siobhan Burns

**Affiliations:** Royal Free and University College, UK

Response to Point 1:

 We agree with the reviewer that bone marrow-derived dendritic cells (BMDC), while widely adopted, represent an imperfect model of in vivo counterparts.  The reviewer makes an important point about whether absence of γc may alter the differentiation of bone marrow cells. In the original manuscript we present evidence that γc-deficient BMDC generated using the protocol described are phenotypically comparable with wild type (WT) controls with respect to MHCII expression and upregulation of the maturation marker CD86 and that several dendritic cell-associated functions, such as antigen uptake and presentation, are the same between γc-deficient and WT BMDC.

 We have additional data to show that γc-deficient and WT BMDC are not different in expression of the DC surface marker CD11c or the maturation marker CD80 (new Figure S2C,E). We did not specifically analyse CD11b subsets within our cultures (as done in the Helft et al manuscript highlighted by the reviewer) to define BMDC from macrophages but we did not see any significant differences in expression of CD80 and CD86 in CD11c+ cells which would be predicted if the proportions of BMDC and macrophages were altered  (as these were specifically identified as markers poorly expressed in bone marrow derived macrophages). Furthermore, we did not see any significant differences in release of the proinflammatory cytokines IL-6 or TNF-a following stimulation with TLR ligands including CpG (which was also shown by Helft et al to differ between BMDC and macrophages; new Figure S2K). Thus, while we cannot exclude minor differences, our data indicate that the phenotype of cells generated from γc-deficient bone marrow using GM-CSF are broadly comparable with WT control.

 In support of our in vitro differentiation data, we also observed that the ex-vivo phenotype of splenic DC was comparable between yc- deficient and WT mice (Figure S1 A,B,C). While there were significantly fewer CD11c+ DCs in the spleens of yc- deficient mice when compared to WT controls (p<0.05), as previously described for other lymphopenic mouse models (Asli B, Lantz O, DiSanto JP, Saeland S, Geissmann F. Roles of lymphoid cells in the differentiation of Langerhans dendritic cells in mice. Immunobiology. 2004;209(1-2):209-21; PMID: 15481155), within the CD11c-enriched fraction, WT and yc/Rag 2-/- mice had similar frequencies of CD11c+ CD11b+ and CD11c+ CD8α+ conventional DCs. CD11c+ B220+ plasmacytoid DC were also present in comparable numbers in the spleens of WT and yc/Rag 2-/- mice. Thus, these results suggest that although required for DC development in vivo, yc is probably dispensable for DC differentiation.

 For this study, bone-marrow (BM) cells were extracted from the femur and tibia of mice. BM-derived DCs were grown from BM cells cultured over 7 days in RPMI medium 1640 supplemented with 10% fetal bovine serum (FBS) and 1% Penicillin/Streptomycin (Gibco) in the presence of 20 ng/ml GM-CSF (BioSource). For all experiments, BM-derived DCs were CD11c selected using magnetic bead separation (Miltenyi Biotech). To induce DC activation, CD11c+ DCs were matured overnight with 100ng/ml LPS (Sigma). This detail has been added to the methods section for clarity.

 Response to Point 2:

 Raw data from a representative example is shown in the original manuscript in supplemental data (now Figure S4C). Addition of IL-15Ra blocking antibody reduced the ability of WT BMDC to induce T-cell proliferation, as assessed by flow cytometry measurement of CFSE dilution, from 54% to 37% at maximal antibody dose (10mcg/ml).  The level of proliferation induced by WT BMDC in the presence of IL-15 blockade was similar to the level of T-cell proliferation induced by γc-/- DC without blockade (37%), suggesting that the defect of T-cell priming seen with γc-/- DC was almost entirely attributable to defective IL-15 transpresentation. Furthermore, there was no real change in T-cell proliferation when γc-/- DC were co-cultured with anti-IL15Ra blocking antibody (reduced from 37% to 36%) indicating that the effect seen in WT cells was specific.

 With respect to the relative importance of IL-15 and IL-2 trans presentation, we tested this experimentally in BMDC: T-cell coculture assays in which we reasoned that BMDC would be transpresenting IL-15 or IL-2 to induce phosphorylation of STAT5 in T-cells. We observed complete abrogation of T-cell pSTAT if we added anti-IL15Ra blocking antibody to the co-culture (Figure 1D) but no change in T-cell pSTAT5 using anti-IL2Ra blockade (Figure S4A,B). This led us to conclude that ‘STAT5 activation occurs primarily through IL-15 transpresentation during DC-mediated priming of naïve CD4+ T cells in our experimental system’. We have added the following sentence to the discussion (end of paragraph2):

 ‘Although it has been previously been shown that blockade of the DC IL-2Ra reduces T-cell activation (Wuest et al, ref 14), we were unable to demonstrate a contribution of DC-mediated IL-2Ra transpresentation in our system leading us to conclude that IL-15 is the major cytokine transpresented by DC for T-cell priming.’
